# Oxysterol signaling in the central nervous system: cellular mechanisms and implications for neurodegeneration

**DOI:** 10.3389/fnmol.2026.1709065

**Published:** 2026-02-12

**Authors:** Raphael P. Ricci, Cheng Xiang Foo, Katharina Ronacher, Carlie L. Cullen

**Affiliations:** 1 Glial Neurobiology, Cognition and Behaviour Research Laboratory, Faculty of Health, Medicine and Behavioural Sciences, Mater Research Institute, The University of Queensland, Brisbane, QLD, Australia; 2 Infection, Immunity and Metabolism Laboratory, Faculty of Health, Medicine and Behavioural Sciences, Mater Research Institute, The University of Queensland, Brisbane, QLD, Australia

**Keywords:** central nervous system, glia, neurodegeneration, neuroinflammation, oxysterols

## Abstract

Oxysterols, oxidized derivatives of cholesterol, are key modulators of central nervous system (CNS) function. Acting as both signaling molecules and cytotoxic mediators, oxysterols can influence glial activity, neuroinflammation and myelination. Glial cells (astrocytes, oligodendrocytes, and microglia) maintain neuronal homeostasis, support synaptic plasticity and mediate CNS repair. Dysregulated glial function contributes to chronic neuroinflammation, a hallmark of neurodegenerative and neuropsychiatric disorders such as Alzheimer’s disease, Parkinson’s disease and multiple sclerosis. In this review article, we discuss the roles of oxysterols in the CNS with a particular focus on their impact on glial cells, neuroinflammation, myelination and neural circuit function. Therapeutically targeting oxysterol metabolism may mitigate oxidative stress, limit apoptosis, enhance glial health and influence myelin repair. Elucidating oxysterol signaling in glia and neurons provides critical insight into neural circuit regulation and identifies promising strategies for treating demyelinating, neurodegenerative and neuropsychiatric disorders.

## 1 Introduction

Ubiquitously present throughout the body, cholesterol is an essential lipid that has a wide variety of physiological functions. Cholesterol is water insoluble and as such, needs to be bound to complex lipoprotein particles to enter the circulation and travel throughout the body. Low-density lipoproteins (LDL) are the principal carrier of cholesterol from the liver to other tissue via LDL receptors. While high-density lipoproteins (HDL) may be involved in reverse transport from other tissue to the liver (Feingold, 2000). As a major component of the cell membrane, cholesterol helps regulate both integrity and fluidity of the cell. Importantly, cholesterol is also a precursor for a variety of bioactive molecules including vitamin D; the five classes of steroid hormones - glucocorticoids, mineralocorticoids, androgens, estrogens and progesterone; and oxysterols (Wolf, 1999; Gill et al., 2008; Hannich et al., 2011; Miller and Auchus, 2011; Zhang L. et al., 2024). The role that vitamin D and steroid hormones play in facilitating central nervous system (CNS) function has been well documented [see (DeLuca et al., 2013; Lebedev et al., 2024; Sailike et al., 2024) for comprehensive reviews], but the role of oxysterols in neurophysiology remains comparatively underexplored.

Endogenously, oxysterols are formed from the oxidization of cholesterol by either non-enzymatic interactions with reactive oxygen species (ROS); or by enzymatic reactions involving cytochrome P450 enzymes or cholesterol-25-hydroxylase (CH25H). These reactions produce various bioactive oxysterols, including 24S-hydroxycholesterol (24S-OHC), 25-hydroxycholesterol (25-OHC), 26-hydroxycholesterol (26-OHC) and 27-hydroxycholesterol (27-OHC) which are the primary bioactive oxysterols in the brain. Through a wide range of cellular and biochemical mechanistic pathways, these oxysterols have been implicated in cell metabolism, infection, immunity and disease pathology – including neuropsychiatric and neurodegenerative disorders (summarized in Table 1) (Vejux et al., 2007; Töröcsik et al., 2009; Yutuc et al., 2020; Foo et al., 2023). In this review, we explore oxysterol signaling in neurons and glial cells and how these cellular mechanisms may influence brain function. We also explore how altered oxysterol signaling may contribute to the pathophysiology of neurodegenerative disorders.

**Table 1:** Oxysterols in disease pathophysiology.

Disease	Oxysterol	Principal cellular targets	Dominant pathophysiological mechanisms	Functional/clinical consequences	References
Alzheimer’s disease (AD)	7β-OHC 7-KC	Neurons, microglia	Lipid peroxidation product; mitochondrial dysfunction; ROS generation; activation of apoptotic and necroptotic pathways	Neuronal loss, neuroinflammation, disease progression	Anderson et al. (2020), Testa et al. (2016)
	24S-OHC	Cortical and hippocampal neurons, astrocytes	Positive allosteric modulation of NMDA receptors; enhanced Ca^2+^ influx; synaptic hyperexcitability; amplification of Aβ-induced toxicity; altered cholesterol turnover	Early synaptic dysfunction, excitotoxic vulnerability, cognitive decline	Linsenbardt et al. (2014), Sun et al. (2016), Gamba et al. (2011)
	27-OHC	Neurons, astrocytes, cerebrovascular endothelium	BBB influx of peripheral sterols; disruption of cholesterol homeostasis; pro-inflammatory signaling; interference with estrogen receptor signaling	Accelerated neurodegeneration, vascular contributions to cognitive impairment	Björkhem et al. (2009), Heverin et al. (2005)
Multiple sclerosis (MS)	7β-OHC 7-KC	Oligodendrocytes, microglia	Oxi-apoptophagy; mitochondrial failure; membrane destabilization; inflammasome activation	Demyelination, axonal degeneration	Nury et al. (2015), Vejux et al. (2021)
	25-OHC	Oligodendrocytes, microglia	NLRP3 inflammasome activation; caspase-1–dependent cell death; suppression of OPC differentiation	Failed remyelination, chronic lesion formation	Jang et al., 2016, Romero et al., 2024
	24S-OHC	Neurons, oligodendrocyte lineage cells	Altered LXR signaling; dysregulated cholesterol efflux; context-dependent effects on myelin repair	Impaired remyelination or compensatory repair depending on disease stage	Meffre et al., 2015, Vejux et al., 2021
Parkinson’s disease (PD)	7β-OHC 7-KC	Dopaminergic neurons (SNpc)	Mitochondrial complex I inhibition; oxidative stress; lipid raft disruption	Nigrostriatal degeneration, motor impairment	Anderson et al. (2020), Gamba et al. (2019)
	24S-OHC	Neurons, astrocytes	Altered brain cholesterol turnover, neurodegeneration	Progressive motor symptoms, dopaminergic neuron loss	Leoni et al. (2005), Papassotiropoulos et al. (2002)
	27-OHC	Dopaminergic neurons, astrocytes	Mitochondrial stress; pro-inflammatory glial activation; disruption of dopamine signaling	Progressive motor and non-motor symptoms	Björkhem et al. (2009), Testa et al. (2016)
Psychiatric disorders (e.g., schizophrenia, depression)	7β-OHC 7-KC	Neurons, glia	Oxidative stress, lipid peroxidation, membrane dysfunction	Cognitive and mood disturbances, altered synaptic function	Pfrieger and Ungerer (2011)
	24S-OHC	Cortical pyramidal neurons, interneurons	Modulation of NMDA receptor function; altered excitation–inhibition balance; impaired synaptic plasticity	Cognitive dysfunction, altered network oscillations	Paul et al. (2013), Sun et al. (2016)
	27-OHC	Limbic neurons, astrocytes	Neuroinflammatory priming; disruption of monoaminergic signaling; oxidative stress	Mood dysregulation, affective symptoms	Segatto et al. (2019); Romero et al. (2024)

Conceptual takeaway: Disease pathophysiology reflects both oxysterol-specific signaling properties and selective cellular vulnerability. Enzymatically generated oxysterols (e.g., 24S-OHC) exert context-dependent neuromodulatory effects, whereas oxidation-derived species (e.g., 7β-OHC and 7-KC) consistently drive mitochondrial dysfunction, inflammation, and cell death across disorders.

## 2 Oxysterol synthesis in the CNS

**Figure 1:**
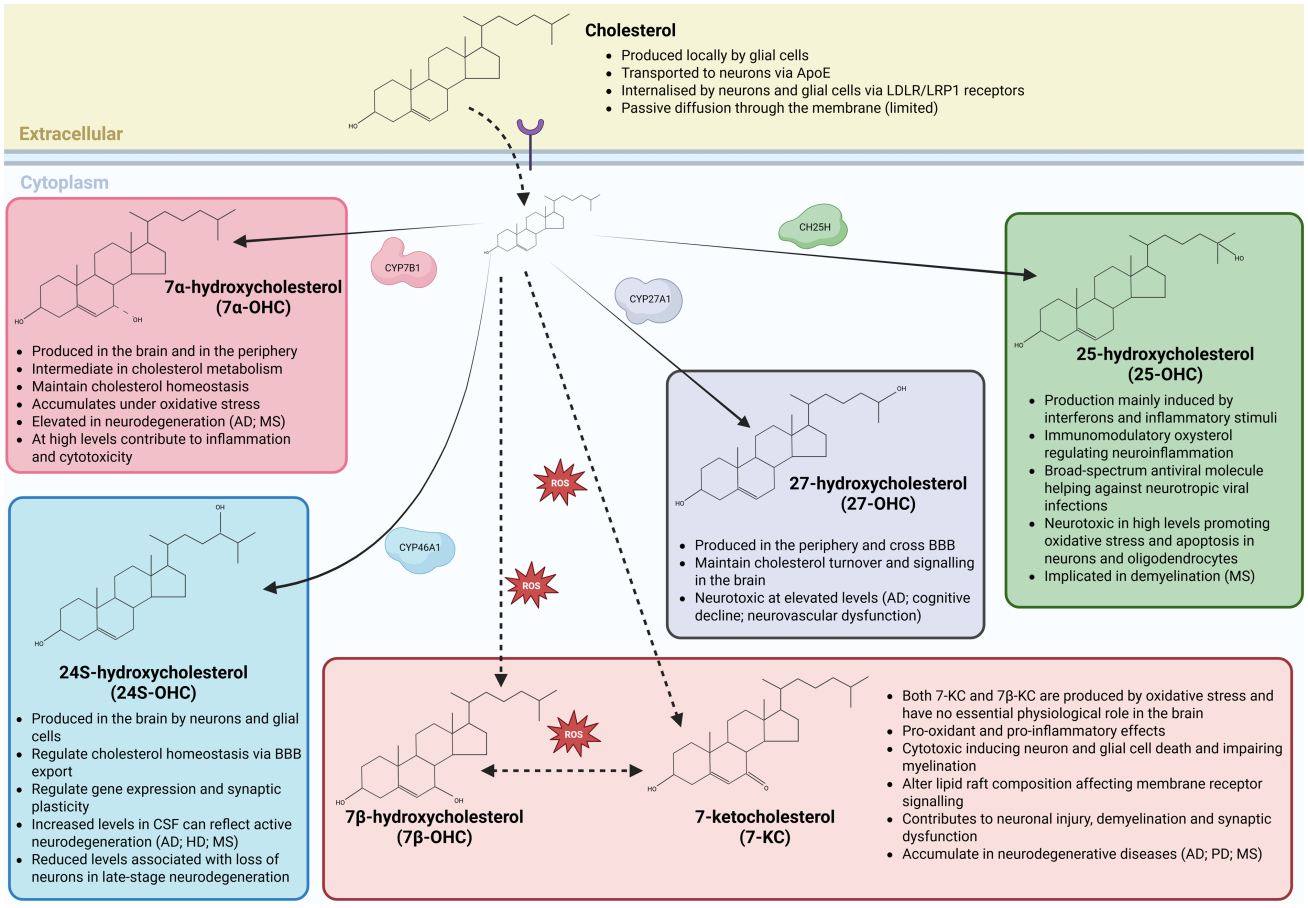
Brain oxysterols: synthesis, transport, and relevance to neuroinflammation and neurodegeneration. Schematic overview of cholesterol metabolism in the central nervous system and the synthesis of major brain-relevant oxysterols through enzymatic and non-enzymatic pathways. Cholesterol is synthesized locally by glial cells and delivered to neurons predominantly via apolipoprotein E (ApoE)–containing lipoproteins, followed by uptake through LDL receptor (LDLR) and LRP1–mediated endocytosis, with limited passive diffusion across membranes. Within neural cells, cholesterol is enzymatically converted into distinct oxysterols with specialized functions. CYP7B1 generates 7α-hydroxycholesterol (7α-OHC), an intermediate in cholesterol metabolism that contributes to cholesterol homeostasis but accumulates under oxidative stress and can promote inflammation and cytotoxicity at elevated levels. Neuron- and glia-expressed CYP46A1 produces 24S-hydroxycholesterol (24S-OHC), the principal brain-derived oxysterol responsible for cholesterol elimination across the blood–brain barrier (BBB) and for modulating gene expression and synaptic plasticity; altered cerebrospinal fluid levels reflect neurodegenerative activity. CYP27A1 generates 27-hydroxycholesterol (27-OHC) primarily in the periphery, which can cross the BBB to influence brain cholesterol turnover and signaling, but becomes neurotoxic at high concentrations (Heverin et al., 2005). Inflammatory cues induce cholesterol-25-hydroxylase (CH25H) to produce 25-hydroxycholesterol (25-OHC), an immunomodulatory oxysterol with antiviral properties that can exacerbate oxidative stress, apoptosis, and demyelination when dysregulated. In parallel, reactive oxygen species (ROS) drive non-enzymatic oxidation of cholesterol to form 7β-hydroxycholesterol (7β-OHC) and 7-ketocholesterol (7-KC), which lack essential physiological roles in the brain and exert pro-oxidant, pro-inflammatory and cytotoxic effects on neurons and glial cells, disrupt lipid raft organization and impair myelination. Accumulation or imbalance of these oxysterols is linked to neurodegenerative and neuroinflammatory disorders, including Alzheimer’s disease, Parkinson’s disease, Huntington’s disease and multiple sclerosis, underscoring the tight coupling between cholesterol metabolism, oxidative stress, and brain pathology.

Brain-derived 24S-OHC is generated by both neurons and glial cells via cholesterol hydroxylation by cytochrome P450 family 46A1 (CYP46A1; also known as cholesterol 24-hydroxylase) enzyme (Figure 1), and facilitates cholesterol efflux across the blood–brain barrier while also functioning as a key signaling molecule influencing gene expression and synaptic plasticity (Lund et al., 1999; Brachet et al., 2015; Chen et al., 2018; Korinek et al., 2020). The concentration of 24S-OHC within cerebrospinal fluid is associated with neuronal integrity across a range of neurodegenerative diseases (Table 1), with concentrations above 2–10 ng / mL typically indicating increased flux out of degenerating neurons (Leoni et al., 2005). In contrast, 25-OHC, produced via cholesterol hydroxylation by cholesterol 25-hydroxylase (CH25H) enzyme in response to interferons and inflammatory cues (Figure 1), links innate immune activation to cholesterol metabolism, exerting potent immunomodulatory and antiviral effects that may become cytotoxic under chronic neuroinflammatory conditions (Dietschy and Turley, 2004; Bauman et al., 2009; Cyster et al., 2014; Son et al., 2023). Peripherally derived 26- and 27-OHC are generated via cholesterol hydroxylation by cytochrome P450 family 27A1 (CYP27A1; also known as sterol 27-hydroxylase) enzyme (Figure 1).

Capable of crossing the blood–brain barrier, both 26- and 27-OHC provide a conduit through which systemic lipid metabolism impacts the brain. More specifically, the accumulation of 26- and 27-OHC within the brain can lead to oxidative stress, neurovascular dysfunction and cognitive decline (Diestel et al., 2003; Ma et al., 2010; Ong et al., 2010; Jeitner et al., 2011). Additional oxysterols, including enzymatically generated 7α-hydroxycholesterol (7α-OHC) and oxidative stress–derived 7β-hydroxycholesterol (7β-OHC) and 7-ketocholesterol (7-KC) (Figure 1), further exemplify the dual nature of oxysterol signaling by acting as intermediates of cholesterol turnover under physiological conditions but amplifying pro-inflammatory, pro-oxidant and cytotoxic pathways during disease (Wnętrzak et al., 2022; Véjux and Lizard, 2009; Mahalakshmi et al., 2021; Radhakrishnan et al., 2025). Together, these oxysterols engage shared receptor systems, including LXRs, G protein-coupled receptor 17 (Gpr17) and G protein-coupled receptor 183 (Gpr183; also known as Epstein–Barr virus-induced receptor 2—Ebi2) across neurons and glial cells, positioning oxysterol signaling as a central integrator of metabolic, immune, and degenerative processes in the healthy and diseased brain (Table 2).

**Table 2:** Overview of oxysterol sources and role in the CNS.

Oxysterol	Primary source/production	Enzymes	Key receptors	Protective/homeostatic roles	Toxic/pathological effects in the CNS	References
Cholesterol	Produced locally by glia Transported to neurons via ApoE Internalised via LDLR/LRP1.	Oxysterol precursor	LDLR, LRP1	Essential for myelin, membranes, synaptic function.	Excess levels can undergo oxidation to toxic oxysterols.	Dietschy and Turley (2004), Pfrieger and Ungerer (2011)
24S-hydroxycholesterol (24S-OHC)	Produced in the brain by neurons and glial cells Exported across BBB.	CYP46A1	LXRα/β; NMDA receptors	Maintains cholesterol turnover and homeostasis Regulates gene expression and synaptic plasticity.	Elevated CSF levels reflect active neurodegeneration (AD, HD, MS) Reduced levels linked to neuronal loss in late-stage disease.	Lund et al. (1999), Björkhem et al. (2009), Testa et al. (2016)
7α-hydroxycholesterol (7α-OHC)	Produced in brain and periphery Intermediate in cholesterol metabolism Increases during oxidative stress.	CYP7B1	LXRα/β	Contributes to cholesterol homeostasis.	High levels promote inflammation and cytotoxicity Accumulates in AD and MS.	Russell (2003), Björkhem and Meaney (2004)
27-hydroxycholesterol (27-OHC)	Produced peripherally Crosses BBB into brain.	CYP27A1	LXRα/β; ERα	Maintains cholesterol turnover and signaling.	Neurotoxic at high levels Associated with AD, cognitive decline, neurovascular dysfunction.	Heverin et al. (2005), Björkhem et al. (2009), Hughes et al. (2013)
25-hydroxycholesterol (25-OHC)	Mainly induced by interferons and inflammatory stimuli.	CH25H	LXRα/β	Immunomodulatory oxysterol Broad-spectrum antiviral molecule (neurotropic viruses).	Promotes oxidative stress, apoptosis, and neuronal/oligodendrocyte injury Implicated in demyelination (MS).	Bauman et al. (2009), Cyster et al. (2014)
7β-hydroxycholesterol (7β-OHC)	Formed via oxidative stress.	Non-enzymatic	Interacts with a variety of cellular receptors and pathways to exert its effects	Potential antiviral properties, but these effects are often overshadowed by its cytotoxicity, especially in severe infections like COVID-19.	Strong pro-oxidant and pro-inflammatory actions Cytotoxic to neurons and glia; impairs myelination Accumulates in AD, PD, MS.	Poli et al. (2013), Testa et al. (2016)
7-ketocholesterol (7-KC)	Formed via oxidative stress.	Non-enzymatic	Interacts with several established nuclear receptors and other protein targets, particularly microglia	Primarily a toxic oxysterol with no known beneficial physiological function Weak antiviral activity in specific contexts, these potential benefits are generally counterbalanced by its potent cytotoxicity.	Potent pro-oxidant/pro-inflammatory oxysterol Alters membrane signaling and lipid raft composition Drives neuronal injury, demyelination, synaptic dysfunction Accumulates in AD, PD, MS.	Véjux and Lizard (2009), Anderson et al. (2020), Testa et al. (2016)

Conceptual takeaway: Oxysterols act as context-dependent lipid signals in the CNS, integrating cholesterol metabolism with receptor-mediated transcriptional, synaptic, and immune pathways. Enzymatically produced oxysterols primarily support cholesterol homeostasis and neuronal function, whereas oxysterols generated during inflammation or oxidative stress accumulate to drive neurotoxicity, neuroinflammation, and myelin dysfunction, thereby linking metabolic imbalance to neurodegenerative disease.

## 3 Oxysterols as a mediator of neuronal function

Oxysterols affect neuron and interneuron function by modulating neurotransmitter release, ion channel activity and nuclear receptor signaling while also influencing oxidative stress, mitochondrial health and glia–neuron interactions (Figure 1). Beneficial oxysterols such as 24S-OHC can enhance plasticity, whereas toxic oxysterols such as 7-KC drive dysfunction and neurodegeneration. Oxysterols modulate neurotransmission both acutely through direct allosteric modulation of ionotropic receptors and membrane effects and chronically through transcriptional regulation via LXRs and oxidative stress pathways. Their actions alter the excitatory/inhibitory balance, which is beneficial for plasticity in normal physiology but pathological when dysregulated. Oxysterols can influence both neurotransmission and neuromodulation by binding to specific receptors such as nuclear receptors or G-protein-coupled receptors, thereby modulating ion channel activity, synaptic plasticity, and neuronal excitability. Through these mechanisms, oxysterols act as key modulators of neuronal communication, linking cholesterol metabolism to dynamic regulation of CNS signaling. In addition to their roles in cholesterol homeostasis, oxysterols participate in neurodevelopment, neuroprotection and the regulation of neuroinflammatory responses, positioning them as important mediators of neuronal health and CNS function. Oxysterol signaling can directly regulate neurotransmission. For instance, oxysterols can alter glutamatergic signaling via direct interference in the function of ionotropic glutamate receptors (NMDA, AMPA, and Kainate), altering gamma-aminobutyric acid (GABA) inhibition and via alterations in neurotransmitters systems like acetylcholine, serotonin and dopamine. Glutamatergic signaling can be influenced by oxysterols via direct regulation of NMDA, AMPA and kainate receptor (KAR) function. As such excess oxysterols (e.g., 7-KC, 27-OHC) can cause glutamate excitotoxicity and neuronal injury (Ma et al., 2010). In neurodegenerative disorders such as MS and AD, dysregulated oxysterol signaling may contribute to glutamate excitotoxicity and demyelination (Diestel et al., 2003; Ong et al., 2010; Jeitner et al., 2011).

### 3.1 Oxysterol regulation of ion channels

Oxysterols can modulate the function of ion channels (Bezine et al., 2018b), contributing to membrane fluidity and lipid raft composition, the activity of voltage-gated sodium and potassium channels and by acting as a positive allosteric modulators for ionotropic glutamate receptors (Olsen et al., 2012; Filomenko et al., 2015). By embedding into the cell membrane, oxysterols impact cholesterol–sphingolipid-rich domains (lipid raft organization) which can lead to alterations in the clustering and function of ion channels and receptors (Filomenko et al., 2015). Side-chain oxysterols can alter membrane thickness, leading to lateral expansion of the bilayer and increasing permeability. Such effects can influence protein behavior, potentially affecting receptor or channel localization (Olsen et al., 2012). For instance, 7β-OHC and 7-KC have shown to destabilize lipid rafts, whereas 25-OHC tends to stabilize them (Wnętrzak et al., 2022). Many membrane channels are localized to lipid rafts, including ion channels. These rafts influence channel activity through direct lipid-protein interactions, alterations in bilayer properties and recruitment of scaffold proteins such as caveolin, impacting protein gating, trafficking and localization (Dart, 2010). In neuronal membranes, such rafts help maintain the clustering and function of voltage-gated channels and synaptic receptors (NMDA, AMPA, and KAR). When oxysterols disrupt raft integrity, channels and receptors may become miss-localized causing altered ion gating or synaptic signaling. This miss-localization could result in compromised excitability and synaptic plasticity, thereby increasing neuronal vulnerability.

Oxysterols can modulate calcium and potassium channel activity via direct channel interaction or subunit composition in addition to alterations in lipid rafts (Bezine et al., 2018a; Bezine et al., 2018b). Direct modulation of ion channel activity impacts excitability of pyramidal neurons and interneurons increasing calcium influx resulting in hyperexcitability or excitotoxicity (Verma et al., 2022). Oxysterols can influence cytoplasmic Ca^2+^ levels by binding directly to the channel-forming subunits of calcium channels, thereby modulating their gating or electrophysiological properties (Mackrill, 2011). Disruption of their function can lead to altered firing patterns and excitability in neurons. In cortical pyramidal neurons, the activity of large conductance calcium channels at the soma and dendrites regulates action potential repolarization and dendritic spike repolarization, respectively (Bock and Stuart, 2016). Voltage-gated potassium (Kv) channels, such as the Kv3 subfamily can also be influenced by oxysterols. Kv channels are essential for fast-frequency firing in inhibitory interneurons. Alterations to the structure or function of these channels can lead to an imbalance of inhibitory and excitatory neurotransmission, affecting neuronal excitability (Bezine et al., 2018a). Inhibitory interneurons rely on Kv3 channels for fast firing and are particularly sensitive to disruptions in ion channel function. Oxysterol-induced alterations in these channels can impair inhibitory signaling, leading to excitation and inhibition imbalances resulting in potential hyperexcitability (Faulkner et al., 2024).

### 3.2 Oxysterol regulation of ionotropic receptors

Recent work has identified oxysterols as important modulators of ionotropic glutamate receptors (iGluRs), positioning these cholesterol-derived metabolites as regulators of excitatory neurotransmission and synaptic plasticity. Acting as endogenous modulators, oxysterols influence receptor function and neuronal excitability, thereby linking brain cholesterol metabolism to glutamatergic signaling in both physiological and pathological contexts (Linsenbardt et al., 2014; Wei et al., 2019; Tang et al., 2020). Accumulating evidence suggests that these lipid mediators can fine-tune synaptic function through direct and indirect interactions with iGluRs, with potential consequences for cognition and neuropsychiatric disease. Though there is limited evidence for direct modulation of GABAergic receptors, the indirect influence of oxysterols on GABAergic signaling is more compelling. These findings highlight the significance of oxysterols in modulating ionotropic receptors, evidencing their potential as therapeutic targets for neurological disorders.

#### 3.2.1 Oxysterol modulation of NMDA receptors

24S-OHC acts as a positive allosteric modulator of NMDARs, enhancing receptor currents and excitatory drive to ultimately influence excitatory neurotransmission. More specifically, 24S-OHC enhances receptor activity by increasing channel opening probability and current amplitude (Paul et al., 2013; Sun et al., 2016) and reducing endogenous 24S-OHC dampens NMDAR function without impacting homeostatic excitability of hippocampal neurons (Sun et al., 2016). The direct oxysterol-receptor interactions appear to be subunit-dependent with receptors containing the NMDA receptor subunit 2B (NR2B) showing greater sensitivity to potentiation by 24S-OHC (Paul et al., 2013; Wei et al., 2019). As NR2B containing NMDARs are thought to have slower activation/deactivation kinetics, leading to an increase in total Ca^2+^ influx into the cell, it is plausible that potentiation of these receptors by 24S-OHC may promote synaptic plasticity (see Shipton and Paulsen, 2014 a review).

Though increased Ca^2+^ influx can promote synaptic plasticity, chronic elevation may drive excitotoxicity. In epilepsy, enhanced NMDAR activation by oxysterols may promote seizure susceptibility (Hanin et al., 2021; Nishi et al., 2022). Evidence suggests that 25-OHC and 27-OHC also potentiate NMDARs, though less potently than 24S-OHC. NMDA activation by these oxysterols can contribute to excitotoxic cascades when present at high concentrations contributing to cellular dysfunction. 7-KC can also alter NMDA function, however, instead of potentiation it disrupts receptor function indirectly through oxidative stress and lipid raft destabilization (Wheless and Rho, 2025). In AD, elevated 24S-OHC and 27-OHC are linked to early synaptic dysfunction (Papassotiropoulos et al., 2002; Russell, 2003; Merino-Serrais et al., 2018; Wang et al., 2019; Wang et al., 2022). Excessive oxysterol accumulation in AD and MS contribute to overactivation of NMDARs sustaining Ca^2+^ influx for longer activating proteases, ROS generation and mitochondrial dysfunction therefore contributing to neurodegeneration (Gamba et al., 2021).

#### 3.2.2 Oxysterol modulation of AMPA receptors

There is growing evidence that oxysterols also modulate AMPA receptor mediated fast excitatory neurotransmission (Wheless and Rho, 2025). Unlike NMDA receptors, AMPARs are less directly sensitive to oxysterols but 24S-OHC, 25-OHC, and 27-OHC can modulate AMPA receptor kinetics, trafficking and synaptic incorporation (Brachet et al., 2015; Korinek et al., 2020). Thus, enhancing excitatory neurotransmission and plasticity in physiology but promoting excitotoxicity in disease context. Oxysterol-induced AMPAR potentiation may lower seizure threshold and worsen excitotoxic damage in people with epilepsy or following ischaemic injury (Wheless and Rho, 2025). In AD, elevated 24S-OHC and 27-OHC reported to alter AMPAR trafficking and impair memory circuits (Merino-Serrais et al., 2018; Staurenghi et al., 2021; Wang et al., 2022). 24S-OHC may weakly potentiate AMPAR-mediated currents in neurons. 24S-OHC acts indirectly by altering membrane cholesterol composition affecting receptor function and mobility (Martin et al., 2014; Korinek et al., 2020). 25-OHC and 27-OHC are reported to alter AMPAR desensitization kinetics and potentially enhance current amplitude in hippocampal neurons (Martin et al., 2014; Korinek et al., 2020). Meanwhile, 7-KC is associated with reduced AMPAR function, possibly by disrupting receptor trafficking and membrane organization. Synaptic plasticity is affected by AMPA receptors being disrupted by oxysterols, such as 24S-OHC which supports early-phase long-term potentiation (LTP), strengthening excitatory transmission (Martin et al., 2014). Conversely, 7-KC accumulation may impair LTP and favor LTD, contributing to cognitive decline in certain neurological disorders (Nury et al., 2020). By increasing AMPAR-mediated excitatory post-synaptic currents (EPSCs), oxysterols shift the excitation/inhibition balance toward excitation, especially when this occurs alongside NMDAR potentiation. This effect can amplify glutamatergic signaling substantially, and sustained AMPAR potentiation, specifically those that lack the 2A subunit, can lead to excitotoxicity due to Ca^2+^ overload and when sustained for long enough can ultimately lead to neuronal death.

#### 3.2.3 Oxysterol modulation of KA receptors

To date, there is little evidence indicating that oxysterols act as direct allosteric modulators of KARs. However, oxysterols influence KARs indirectly through the membrane lipid environment (Korinek et al., 2020; Wnętrzak et al., 2022; Wnętrzak et al., 2024). KARs are highly sensitive to cholesterol and lipid raft composition. Oxysterols such as 24S-OHC, 25-OHC, 27-OHC and 7-KC intercalate into membranes and alter receptor conformation and trafficking. In neurodegenerative diseases such as AD, amyotrophic lateral sclerosis (ALS) and MS elevated oxysterols disrupt lipid rafts, impairing KAR stability at synapses and contribute to synaptic failure (Grassi et al., 2020; Vallés and Barrantes, 2022; Wnętrzak et al., 2022). KARs can also be disrupted via crosstalk with NMDA and AMPA receptors. For example, KARs share synaptic signaling domains and oxysterol-driven enhancement of glutamatergic tone via NMDA/AMPA receptors indirectly changes KAR activation (Vallés and Barrantes, 2022). Oxysterols could also alter KARs presynaptic modulation by regulating glutamate release probability at excitatory terminals (Lauri et al., 2005). By changing membrane cholesterol and calcium dynamics, oxysterols may increase presynaptic glutamate release via KAR activation amplifying excitation. In epilepsy, dysregulated KAR signaling - already implicated in seizure susceptibility - may be worsened by oxysterol-induced increases in presynaptic glutamate release (Rodríguez-Moreno and Sihra, 2013; Fritsch et al., 2014). KARs contribute to slower EPSCs and metabotropic-like signaling (Lauri et al., 2005). Oxysterols that destabilize cholesterol-rich domains such as 7-KC, can impair KAR clustering at postsynaptic densities, weakening KAR-mediated excitatory currents (Farooqui, 2009; Wnętrzak et al., 2022).

KARs are expressed on both excitatory and inhibitory neurons and as such play a role in maintaining the excitation/inhibition balance within the brain. Oxysterol-driven disruption of KAR function could weaken inhibitory interneuron regulation, tipping networks toward hyperexcitability. KARs contribute to forms of LTP/long term depression (LTD) ratio distinct from NMDARs. Oxysterol-induced shifts in receptor trafficking may bias synapses toward excitotoxic plasticity. KARs – especially those containing Glutamate receptor ionotropic kainate 4 and 5 (GluK4/GluK5), mediate oligodendrocyte and neuronal vulnerability (Lerma and Marques, 2013). Oxysterol accumulation in demyelinated lesions, notably 7-KC and 24S-OHC may exacerbate KAR-mediated excitotoxicity in oligodendrocytes (Matejuk et al., 2025). Oxysterols modulation of KARs indirectly by altering membrane cholesterol dynamics, receptor trafficking and glutamate release shapes excitatory/inhibitory neurotransmission balance potentially contributing to excitotoxicity in epilepsy and neurodegeneration. In summary, iGlu (NMDA, AMPA and KARs) potentiation by oxysterols facilitates LTP strengthening excitatory synapses linking cholesterol metabolism to learning and memory. By boosting glutamatergic drive, oxysterols shift the excitation/inhibition balance toward excitation, contributing to excitotoxicity.

#### 3.2.4 Oxysterol modulation of GABA receptors

Excitation can also be exacerbated by reduced GABAergic signaling (Shore et al., 2020; Jiménez-Balado and Eich, 2021). Disturbances in oxysterol signaling may shift the excitation/inhibition balance, a feature implicated in many neurological disorders including epilepsy and schizophrenia (Shore et al., 2020; Santoro et al., 2024; Crotchett et al., 2025). Oxysterol effects on GABAergic interneurons involve redox balance, cholesterol metabolism and mitochondrial signaling (Poli et al., 2013; Calvo and González, 2016; Beltrán González et al., 2020). Oxysterols like 7-KC promote oxidative stress and ROS generation. ROS can modify GABAA_AA receptor subunits or presynaptic GABA release machinery, impairing synaptic inhibition (Beltrán González et al., 2020). Gamma-Amino-Butyric Acid-A (GABA_A_) receptor function is highly dependent on membrane cholesterol (Sooksawate and Simmonds, 1998, 2001; Meza et al., 2020; Yuan et al., 2025). Oxysterols can alter GABAergic inhibition via cholesterol depletion and substitution effects (Sooksawate and Simmonds, 1998; Meza et al., 2020). Pathological oxysterols can displace or promote an increase in cholesterol oxidization, leading to reduced GABA_A_ receptor surface stability and faster receptor internalization reducing inhibition (Sooksawate and Simmonds, 1998; Meza et al., 2020; Jiménez-Balado and Eich, 2021). Inhibition can be classified in tonic and basic (Farrant and Nusser, 2005). Tonic inhibition is a persistent, low-level suppression of neuronal excitability mediated by extra synaptic GABA_A_ receptors that respond to ambient levels of the neurotransmitter GABA in the brain. It provides a stable, baseline inhibitory tone that sets the overall excitability of neurons mediated by extra-synaptic GABA_A_. It sets neuronal excitability thresholds and contributes to network stability. Certain oxysterols may reduce inhibitory tone by destabilizing GABA_A_ receptor function (Calvo and González, 2016; Mele et al., 2019; Michałowski et al., 2025). Tonic GABA currents depend on the stable surface expression of extra-synaptic receptors in cholesterol-rich microdomains (Carver and Reddy, 2013). Pathological oxysterols like 7-KC or 25-OHC can disrupt lipid raft integrity and reduce surface stability of extra-synaptic GABA_A_ receptors (Carver and Reddy, 2013). Disruption of lipid raft integrity can weaken tonic inhibition and increase susceptibility to hyperexcitability. Tonic currents are also sensitive to redox signaling (Calvo and González, 2016). Tonic GABA_A_ receptors are especially redox-sensitive therefore ROS from oxysterols, e.g., 7-KC may reduce their efficacy (Calvo and González, 2016; Anderson et al., 2020; Beltrán González et al., 2020). GABA_A_ receptors are speculated to also be directly modulated by oxysterols. However, to this date there is no direct evidence indicating direct modulation. Altered tonic network excitability can enhance the amplitude and/or prolong the decay of phasic inhibitory postsynaptic currents (IPSCs) (Ataka and Gu, 2006; Labarrera et al., 2013). Oxysterols can also indirectly influence astrocytic cholesterol and GABA metabolism (Labarrera et al., 2013; Mahalakshmi et al., 2021; Liu et al., 2022; Radhakrishnan et al., 2025; Yuan et al., 2025). Elevated oxysterols, especially 7-KC, can impair astrocyte function and glutamate/GABA balance reducing extracellular GABA and weakening tonic inhibition (Mahalakshmi et al., 2021; Radhakrishnan et al., 2025). Conversely, 24S-OHC is produced largely by neurons and may help maintain inhibitory tone under physiological conditions (Leoni and Caccia, 2013; Vitali et al., 2014; Noguchi et al., 2015). Other oxysterols, e.g., 7-KC and 25-OHC can destabilize membrane lipid rafts, altering GABA_A_ receptor trafficking or clustering at synapses (Radhakrishnan et al., 2007; Mahalakshmi et al., 2021; Radhakrishnan et al., 2025). Receptor trafficking and clustering may reduce phasic inhibition efficiency. Phasic inhibition is the rapid, short-lived inhibition of neuronal activity mediated by GABAergic neurotransmitters released from presynaptic terminals that activate GABA_A_ receptors on the postsynaptic membrane (Farrant and Nusser, 2005). In contrast to tonic inhibition, which is a persistent inhibition caused by extracellular GABA activating extra synaptic receptors, phasic inhibition is triggered by discrete synaptic events (Farrant and Nusser, 2005). In other words, it is a fast synaptic IPSCs via clustered GABA_A_ receptors. Since oxysterols strongly enhance glutamatergic signaling, relative dampening or enhancing of GABAergic phasic inhibition directly shifts the excitation/inhibition balance. For example, 24S-OHC might strengthen inhibition exerting a protective effect and 7-KC might weaken inhibition and be pro-excitotoxic. This imbalance (increased glutamatergic drive, reduced inhibitory control) contributes to hyperexcitability and excitotoxicity.

Both ionotropic glutamate and GABA receptors can be indirectly modulated by synaptic vesicle dynamics and altered release probability (Fattorini et al., 2019; Wallace and Sabatini, 2023). Oxysterols can incorporate into membranes, altering lipid raft structure and thereby modulating SNARE complex function and vesicle release probability, affecting glutamate and GABA release (Chamberlain et al., 2001; Salaün et al., 2005; Grassi et al., 2020). AMPA receptors alternate between synaptic and extra-synaptic membranes as part of synaptic plasticity (LTP/LTD). Oxysterols modify lipid raft structure and membrane fluidity, altering AMPAR dynamics and clustering at postsynaptic densities (Filomenko et al., 2015; Wnętrzak et al., 2022). It also modulates receptor endocytosis/exocytosis during activity-dependent plasticity (Kasimov et al., 2017; Petrov, 2024a). As a result, oxysterols can bias plasticity toward potentiation or depression depending on which oxysterol predominates. 7-KC has been shown to impair vesicular cycling and contribute to synaptic dysfunction in neurodegeneration (Mahalakshmi et al., 2021; Shin et al., 2024). Physiological oxysterols, e.g., 24S-OHC enhance tonic and stabilise inhibition by positively modulating *δ*-subunit–containing GABA_A_ receptors (Leoni and Caccia, 2013; Vitali et al., 2014; Noguchi et al., 2015). Pathological oxysterols, e.g., 7-KC, 25-OHC reduce tonic inhibition via membrane disruption, receptor destabilization, oxidative stress and impaired astrocytic support (Mahalakshmi et al., 2021; Radhakrishnan et al., 2025). Receptor balance is crucial because tonic inhibition is a major controller of excitability therefore oxysterol shift can drive neural networks toward seizure susceptibility or neuroprotection depending on the context. Oxysterols influence phasic inhibition mainly by directly modulating GABA_A_ receptor activity, altering receptor stability in cholesterol-rich membranes and indirectly through oxidative stress effects. Oxysterols like 24S-OHC tend to enhance phasic inhibition, whereas oxysterols such as 7-ketocholesterol (increased in pathological context) reduces it, contributing to network hyperexcitability and possibly seizures or neurodegeneration (Gamba et al., 2021).

### 3.3 Oxysterol modulation of cholinergic, serotonergic and dopaminergic systems

Neuromodulators are chemical signals that regulate neuronal activity and synaptic transmission over longer timescales and broader spatial domains than classical neurotransmitters. Neuromodulators such as dopamine, serotonin and certain neuropeptides can fine-tune network excitability, synaptic plasticity and overall information processing in the CNS. Oxysterols can influence the cholinergic, serotonergic and dopaminergic systems (Ma et al., 2009; Ullrich et al., 2010; Paul et al., 2017; Kosowski et al., 2021), leading to indirect influence on mood, motor control, cognition and consciousness. Cholesterol metabolism is essential for synaptic vesicle dynamics and oxysterols alter membrane lipid composition disrupting vesicle fusion and neurotransmitter release (Petrov, 2024b). As their name suggests, the primary neurotransmitter for cholinergic neurons is acetylcholine. Cholinergic receptors are divided in two mains types, nicotinic receptors (ligand-gated ion channels) and muscarinic receptors (G-protein-coupled receptors) 0.7-KC and 25-OHC impair choline uptake and ACh release by altering presynaptic terminal integrity and mitochondrial function (ATP supply for vesicle cycling) (Ullrich et al., 2010). Oxysterols also modulate cholinergic receptors (Ullrich et al., 2010; Baenziger et al., 2017; Petrov, 2024a). Membrane cholesterol and oxysterols can regulate nicotinic acetylcholine receptors (nAChRs) stability and gating (Baenziger et al., 2017). Oxysterols may reduce receptor function by disturbing lipid rafts where nAChRs cluster (Baenziger et al., 2017). Oxysterols, especially 27-OHC can also interact with estrogen receptors and G protein-coupled receptors (GPCR) signaling indirectly modulating muscarinic acetylcholine receptors (mAChRs) activity and downstream calcium responses (Wang et al., 2021). Critically relevant to diseases such as AD where both cholinergic decline and altered sterol metabolism occur (Auld et al., 2002; Reiss and Voloshyna, 2012; Comaposada-Baró et al., 2023). Cholinergic neuron loss is a hallmark of AD (Auld et al., 2002) and elevated oxysterols such as 24S-OHC, 27-OHC, 7-KC are detected in AD brains and cerebrospinal fluid (Dias et al., 2022). Such oxysterols may exacerbate cholinergic dysfunction via oxidative stress, mitochondrial damage and impaired receptor signaling. In PD, altered oxysterol metabolism may contribute to degeneration in the cholinergic basal forebrain and striatal interneurons worsening motor and cognitive symptoms (Müller and Bohnen, 2013; Gan et al., 2023; Brahmi et al., 2025). Oxysterols can also impair the cholinergic system by disrupting ACh release, destabilizing nicotinic receptor function, interfering with muscarinic signaling pathways, and aggravating neuroinflammation that normally would be modulated by cholinergic anti-inflammatory signaling receptors (Ullrich et al., 2010; Baenziger et al., 2017; Petrov, 2024a). These mechanisms link oxysterols to cholinergic deficits seen in neurodegenerative conditions like AD.

Dopamine synthesis and release can also be altered by oxysterols (Sacchetti et al., 2009; Hennegan et al., 2024). Dopamine neurons are highly sensitive to oxidative stress (Guo et al., 2018; Bej et al., 2024). Oxysterols like 7-KC and 27-OHC cause mitochondrial dysfunction and ROS production, impairing ATP supply and consequently vesicular dopamine release (Dasari et al., 2010; Wang et al., 2016; Anderson et al., 2020; McComb et al., 2021; Vejux et al., 2025). 24S-OHC has been shown to alter synaptic vesicle dynamics indirectly via effects on lipid rafts and calcium influx. Dopamine receptors’ function is also altered by oxysterols. Membrane cholesterol is crucial for GPCR stability, including D1/D2 receptors (Paila and Chattopadhyay, 2009; Kumar and Chattopadhyay, 2021; Christofidi et al., 2025). Excess oxysterols may destabilize receptor conformation disrupting dopamine receptor signaling. 27-OHC has been reported to reduce dopamine signaling (Marwarha et al., 2011) and contribute to learning and memory deficits in rats (Zhang et al., 2015). In PD, dopaminergic neurons in the substantia nigra accumulate oxysterols under oxidative stress (Hwang, 2013; Doria et al., 2016). Oxysterol 7-KC is cytotoxic to dopaminergic neurons and drives degeneration (Wang et al., 2016; Anderson et al., 2020). Dopaminergic disruption by oxysterols is also observed in psychiatric disorders such as schizophrenia (Sun et al., 2021) and depression (Sun et al., 2023). Dysregulated oxysterol metabolism may indirectly modulate mesolimbic dopamine circuits, affecting motivation and reward.

Oxysterols also affect serotonin synthesis and neuron viability (Dodge et al., 2021). Serotonergic neurons in the raphe nuclei are vulnerable to oxidative stress (Zhang C. et al., 2024). 7-KC and 27-OHC increase ER stress and apoptosis in serotonergic neurons potentially decreasing 5-HT availability. Oxysterols also indirectly disrupt serotonin transporter and receptors. Serotonin transporter function depends on lipid raft integrity and oxysterols disruption of lipid rafts alter serotonin reuptake (Daray et al., 2018). Oxysterols can also indirectly modulate serotonin receptors 5-HT1A and 5-HT2A GPCR signaling by disturbing membrane cholesterol balance (Daray et al., 2018). As such, elevated plasma 27-OHC has been associated with major depressive disorder as a result of impaired serotonin signaling, hypothalamic–pituitary–adrenal (HPA) axis dysregulation and hippocampal neurotoxicity (Daray et al., 2018; Sun et al., 2023; Wang et al., 2023). In AD, serotonergic deficits coexist with high oxysterol levels, compounding cognitive and mood symptoms (Testa et al., 2016).

Oxysterols impair cholinergic, dopaminergic and serotonergic function via oxidative stress, vesicle release impairment, receptor destabilization and transporter dysfunction. 7-KC and 27-OHC are largely neurotoxic, while 24S-OHC has dual roles, sometimes protective through LXRs, sometimes harmful at high levels. These mechanisms are implicated in several neurological disorders. Those neurotransmitter systems can also be modulated by oxysterol-driven LXR signaling (Mouzat et al., 2019). LXRs regulate genes for lipid metabolism but also influence dopamine/serotonin turnover and neuroinflammation (Töröcsik et al., 2009; Mouzat et al., 2019). 27-OHC crosses the blood–brain barrier and acts as an endocrine oxysterol, disrupting striatal dopamine and cortical serotonin simultaneously (Zhang et al., 2015; Kim et al., 2022). This may explain links between hypercholesterolemia and risk of mood disorders and PD (Kim et al., 2022; Sun et al., 2023). Cholinergic system function can also be indirectly modulated via LXR activation. Oxysterols like 24S-OHC and 27-OHC are natural LXR ligands. In neurons, LXRs regulate lipid homeostasis, synapse formation, and plasticity. In interneurons, LXR signaling can affect interneuron survival and development (particularly during cortical wiring). LXR regulates genes linked to cholesterol transport (ABCA1, ApoE), neuroinflammation, and synaptic proteins (Cao et al., 2007; Zhang R. et al., 2024). This influences long-term plasticity and homeostasis of neurotransmitter systems.

## 4 Oxidative stress, mitochondrial dysfunction, and lipid peroxidation

Oxysterols can act as pro-oxidant molecules and once formed can amplify oxidative stress. This effect is observed in the form of mitochondrial damage, nicotinamide adenine dinucleotide phosphate (NADPH) oxidase activation and depletion of antioxidants. Some oxysterols - especially 7-KC—disrupt mitochondrial membrane potential, impair electron transport chain activity, and increase ROS leakage (Anderson et al., 2020). Increased oxysterol metabolism can drive a feed-forward cycle of ROS and oxysterol production, further amplifying oxysterol production, causing myelin and neuron damage (Bezine et al., 2018b). Research suggests that certain oxysterols, particularly those produced from non-enzymatic cholesterol oxidation, may be associated with neuroaxonal injury in people with MS. For example, higher levels of 7-KC and 7β-hydroxycholesterol (7β-OHC) are associated with increased levels of serum neurofilament light chain (sNfL, a protein found in the nervous system that is released into the blood and CSF when neurons are damaged commonly used as a marker of neuroaxonal damage) in people with MS. Some studies suggest that oxysterols may be markers of specific disease processes in MS, though more research is needed to fully understand these relationships. Oxysterols can be considered as biomarkers of choice but also as major actors in the pathophysiology of MS (Vejux et al., 2021). 7-KC and 24S-OHC enhance ROS production, leading to oxidative damage in neurons and glial cells. 7-KC and 24S-OHC can activate NADPH oxidases, boosting superoxide production. *In vitro* studies using neurons treated with 24-OHC in the presence of apocynin—a known inhibitor of NADPH oxidase—evidenced NADPH oxidase–mediated ROS generation under 24S-OHC treatment (Testa et al., 2018). 7-KC has shown to reduce mitochondrial membrane potential in oligodendrocytes (Nury et al., 2018), resulting in mitochondrial damage and reduced ATP production (Nury et al., 2018; Anderson et al., 2020). Similarly, 24S-OHC contributes to oxidative stress via increased ROS production, mitochondrial dysfunction and disruption of antioxidant systems. 24S-OHC can negatively affect the activity of mitochondrial complexes, which are a major site of ROS production. The increased oxidative stress and damage caused by high 24S-OHC levels contribute to neuronal cell death and dysfunction (Cigliano et al., 2019). Increased levels of 24S-OHC are a known factor in the development of AD, where it amplifies the neurotoxic action of amyloid-beta (Aβ). The presence of 24-OHC in the close vicinity of amyloid plaques enhance the adhesion of large amounts of Aβ to the plasma membrane of neurons amplifying the neurotoxic action of Aβ locally increasing ROS steady-state levels (Gamba et al., 2011; Gamba et al., 2021). Both 7-KC and 27-OHC have been linked to mitochondrial dysfunction in AD (Gamba et al., 2019). 27-OHC is elevated in AD patients and is associated with cognitive decline (Leoni and Caccia, 2011). Oxysterols can impair mitochondrial function by disrupting the electron transport chain, reducing ATP production, and increasing apoptosis. Neurons, especially fast-spiking interneurons, are highly energy-demanding and mitochondrial stress makes them vulnerable to dysfunction and loss disrupting network synchrony and function leading to neurodegeneration (Hughes et al., 2013). The combined effects of oxidative stress and mitochondrial dysfunction induced by 7-KC and 27-OHC ultimately contribute to myelin damage and neuronal cell death, key aspects of neurodegenerative diseases (Guo et al., 2013; Singh et al., 2019; Wen et al., 2025).

Oxysterols trigger stress kinases (p38, c-Jun N-terminal kinase—JNK) linking oxidative stress to apoptosis (Lordan et al., 2009). Several oxysterols including 24S-OHC, 25-OHC, 27-OHC, 7-KC can activate Mitogen-Activated Protein Kinase (MAPK) signaling pathways (Extracellular Signal-Regulated Kinase - ERK1/2, JNK, and p38) through the generation of ROS, which engage redox-sensitive kinases upstream of MAPK. Oxysterols generate ROS by damaging mitochondria, activating nitric oxide enzymes, inducing endoplasmic reticulum stress and amplifying inflammatory cascades. Additionally, oxysterols can also interact with other receptors such as LXR which converge on MAPK cascades. Oxysterol activation of JNK and p38 through ROS-dependent signaling, endoplasmic reticulum stress–mediated pathways and inflammatory receptor activation is generally associated with the promotion of apoptosis, while ERK activity inhibits apoptosis (Cross et al., 2000). Oxysterol-driven JNK and p38 activation promotes cell death in neurons and glial cells (Harper and LoGrasso, 2001; Choi et al., 2004). Numerous *in vitro* studies have demonstrated modifications in ERK-1/2 activity following exposure to oxysterols (Ares et al., 2000; Yoon et al., 2004; Leonarduzzi et al., 2005) therefore altering endogenous apoptosis inhibition. *In vitro* studies using C6 glioma cells 7β-OH application induced a decrease in ERK-1/2 activity followed by toxicity (Adamczyk et al., 1998). Moreover, administration of 7-KC in Tohoku Hospital Pediatrics-1 (THP-1) cells - a human monocytic leukemia cell line derived from a patient with acute monocytic leukemia—induced ERK-1/2 phosphorylation increasing apoptosis significantly (Berthier et al., 2005). Together, this evidence underscores how oxysterol-driven MAPK signaling can tip the balance toward neurodegeneration, with JNK and p38 activation overriding ERK’s protective effects to promote apoptosis in neurons and glia.

Neurons are inherently susceptible to oxidative stress, partly due to their relatively low levels of antioxidant enzymes such as Glutathione Peroxidase (GPx), Superoxide Dismutase (SOD) and catalase (Dringen, 2000). Oxysterols can consume glutathione (GSH) and inhibit antioxidant enzymes such as glutathione peroxidase and superoxide dismutase. Under oxidative-stress-related conditions, depletion of GSH and impairment of these antioxidant enzymes correlate with neurodegeneration (Testa et al., 2016). 7-KC was linked to increased ROS formation and GSH depletion in neurons, indicating oxidative stress as a key driver of cell damage (Lee et al., 2007). PC12 neuronal-like cells *in vitro* exposed to 7-KC show a decrease in GSH levels, mitochondrial dysfunction, elevated ROS and activation of caspase-3 apoptotic pathways (Han et al., 2007). The data suggest that mature neurons express low levels of Nuclear Factor E2-related Factor 2 (Nrf2) compared to glial cells (Levings et al., 2023). This inherently limited Nrf2 expression may constrain antioxidant responses, making neurons more susceptible to oxidative stress. Therefore, Nrf2 suppression in neurons potentially contributes to neurodegeneration. All those events contribute to oxidative stress exacerbating mitochondrial dysfunction, further impairing energy metabolism and neuron and glia survival.

Oxysterols also contribute to lipid peroxidation resulting in neuron degeneration (Sultana et al., 2013). Oxysterols initiate chain reactions of lipid peroxidation by producing lipid radicals and reactive aldehydes from polyunsaturated fatty acids in membranes, e.g., 4-hydroxynonenal (4-HNE) and malondialdehyde (MDA) (Gęgotek and Skrzydlewska, 2019). A large case–control study found elevated serum MDA levels in people with relapsing–remitting MS (RRMS), particularly during relapse phases. These levels correlated with disability scores (EDSS), with higher MDA detected in people with MS not on disease-modifying therapy (IFN-β) (Ghonimi et al., 2021). A meta-analysis reviewing oxidative stress markers across 31 studies found that MDA levels (and lipid hydroperoxides) in blood and CSF were consistently higher in people with MS compared to healthy controls (Zhang et al., 2020). In neurons, 4-HNE and MDA induce the formation of protein and DNA adducts impairing neuronal function. In AD, elevated 4-HNE adducts accelerate aggregation of tau and amyloid-β followed by synaptic loss (Dalleau et al., 2013; Sultana et al., 2013). In people with MS, lipid aldehyde adducts are found in CSF samples and demyelinated axons and are responsible for exacerbating neurodegeneration (Gonzalo et al., 2012; Zhang et al., 2020). Protein adducts are formed due to binding of 4-HNE and MDA amino acid residues Cys, His and Lys in neuronal proteins such as synaptic and cytoskeleton proteins, ion channels and receptors and mitochondrial enzymes. This leads to synaptic dysfunction, impaired plasticity and vulnerability to excitotoxic cell death of neurons (Neely et al., 1999). DNA adducts are formed when 4-HNE and MDA react with DNA bases resulting in replication error and mutation, activation of DNA damage response and mitochondrial damage. It causes neurons to accumulate DNA lesions and undergo progressive dysfunction (Voulgaridou et al., 2011). Furthermore, lipid peroxidation associated with GSH depletion leads to oxysterol-induced death of neurons via ferroptosis (Ren et al., 2020; Li et al., 2022). Lipid peroxidation also alters membrane fluidity, affecting ion channel function and receptor signaling (Ghosh et al., 1993; Chen and Yu, 1994). Oxidation of lipid rafts and synaptic vesicle membranes alters voltage-gated channels, N-methyl-D-aspartate (NMDA), *α*-amino-3-hydroxy-5-methyl-4-isoxazolepropionic acid receptors (AMPA) and kainate receptor (KAR) localization consequently impairing neuronal function and making neurons vulnerable to excitotoxicity leading to neurodegeneration (Mattson, 1998; Hering et al., 2003; Korinek et al., 2020). Once initiated, lipid peroxidation can further oxidize cholesterol to produce more oxysterols in a feedback loop. These lipid peroxidation products also activate microglia, perpetuating oxidative stress and contributing to chronic neuroinflammation (Farooqui and Farooqui, 2011; Cumaoğlu et al., 2019). Oxysterols both result from and fuel oxidative stress. They damage mitochondria, activate ROS-producing enzymes, and initiate lipid peroxidation, leading to a cascade of events that worsens inflammation and cell death of neurons significantly contributing to neurodegeneration.

## 5 Roles of oxysterols in glial function and neuroinflammation

Glia are the non-neuronal cells of the CNS that provide essential support and regulation for neuronal function. The three major glial cells are astrocytes, oligodendrocytes and microglia. Astrocytes maintain extracellular ion and neurotransmitter homeostasis, regulate blood–brain barrier integrity and provide metabolic support to neurons. Oligodendrocytes generate and maintain myelin sheaths around axons, enabling rapid saltatory conduction and contributing to axonal health. Microglia are the resident immune cells of the CNS, responsible for surveillance, phagocytosis of debris and modulation of inflammatory responses. Together, these glial populations are crucial not only for maintaining CNS homeostasis but also for facilitating development, plasticity and repair. Dysregulation of these glial cell functions can contribute to chronic neuroinflammation, a hallmark of many neurodegenerative and neuropsychiatric disorders. Chronic neuroinflammation is a central feature of several neurodegenerative diseases like AD, PD and MS. Glial cell activation is a key component of neuroinflammation, accompanied by the activation of other cell types such as peripheral immune cells and neurovascular endothelial cells. Given the pro-inflammatory functions of oxysterols in the peripheral organs, it is plausible that oxysterols could be a key driver of neuroinflammation and neuronal damage in the brain. In AD patients, several oxysterols including 27-OHC, 25-OHC, 7α, OHC, 7-KC, 7β-OHC, 5α,6α-epoxycholesterol, 5β,6β-epoxycholesterol, 4α-OHC and 4β-OHC were increased in frontal and occipital cortex, coincided with an increased expression of pro-inflammatory markers such as IL-1β, IL-6, CCL2, MMP-9, and IL-8 (Testa et al., 2016). In addition, in an LPS-mediated neuroinflammation model, the concentration of 25-OHC in the cortex positively correlated with levels TNF, IL-6, and IL-1β (Romero et al., 2024).

### 5.1 The influence of oxysterols on oligodendrocyte lineage cells and myelin

#### 5.1.1 Oxysterol regulation of inflammation in oligodendrocyte lineage cells

In murine oligodendrocytes (158 N), 7KC, 7β-OHC, and 24S-OHC have been identified as potent inducers of oxi-apoptophagy, a form of cell death involving the simultaneous activation of oxidative stress, apoptosis and autophagy pathways (Nury et al., 2015; Leoni et al., 2017). Treatment with these oxysterols resulted in reduced cell proliferation and adhesion, mitochondrial activity and nuclear fragmentation. Induction of apoptosis was characterized by caspase-3 activation, PARP degradation and the downregulation of Bcl-2. Oxidative stress was confirmed by the increased ROS production measured through the elevated concentrations of superoxide anion and hydrogen peroxide, while autophagy was indicated by an increased LC3-II/LC3-I ratio. Additionally, 7-KC has been shown to induce peroxisomal dysfunction, evidenced by the reduced expression of ABCD3 transporter and the activation of pexophagy, as observed through electron microscopy. This dysfunction was associated with an impaired peroxisomal β-oxidation, marked by increased levels of very long-chain fatty acids and the reduced expression of key genes involved in the β-oxidation pathway such as ABCD1, ACOX1 and MFP2 (Nury et al., 2018). In mice, stereotactic administration of 25-OHC into the brain has been shown to drive oligodendrocyte apoptosis in an NLRP3-dependent manner (Jang et al., 2016). Brain regions treated with 25-OHC revealed an increased co-localization of caspase-3 expression with MBP, compared to vehicle-treated controls. Furthermore, the 25-OHC-induced loss of oligodendrocytes was prevented in *Nlrp3*^−/−^ out treated with 25-OHC, highlighting the critical role of the NLRP3 inflammasome in mediating 25-OHC-driven oligodendrocyte death. The pro-apoptotic role of 25-OHC has also been demonstrated in 158 N oligodendrocytes, where it induced apoptosis via increased caspase-3 cleavage activity. Mechanistically, 25-OHC activates the secretory phospholipase A2 type IIA (sPLA2-IIA) promotor and its subsequent expression through the activation of LXRβ and PXR. The activation appears to be dose-dependent, with low dose 25-OHC activating LXRβ signaling, while high doses activate PXR (Trousson et al., 2009). Apart from driving inflammation and cell death, oxysterols have also demonstrated to drive migration. In human MO3.13 oligodendrocytes, administration of 7α,25-OHC led to increased cell migration, an effect that was antagonized by NIBR189 (Velasco-Estevez et al., 2021).

#### 5.1.2 Oxysterols as signaling molecules in the oligodendrocyte lineage

**Figure 2:**
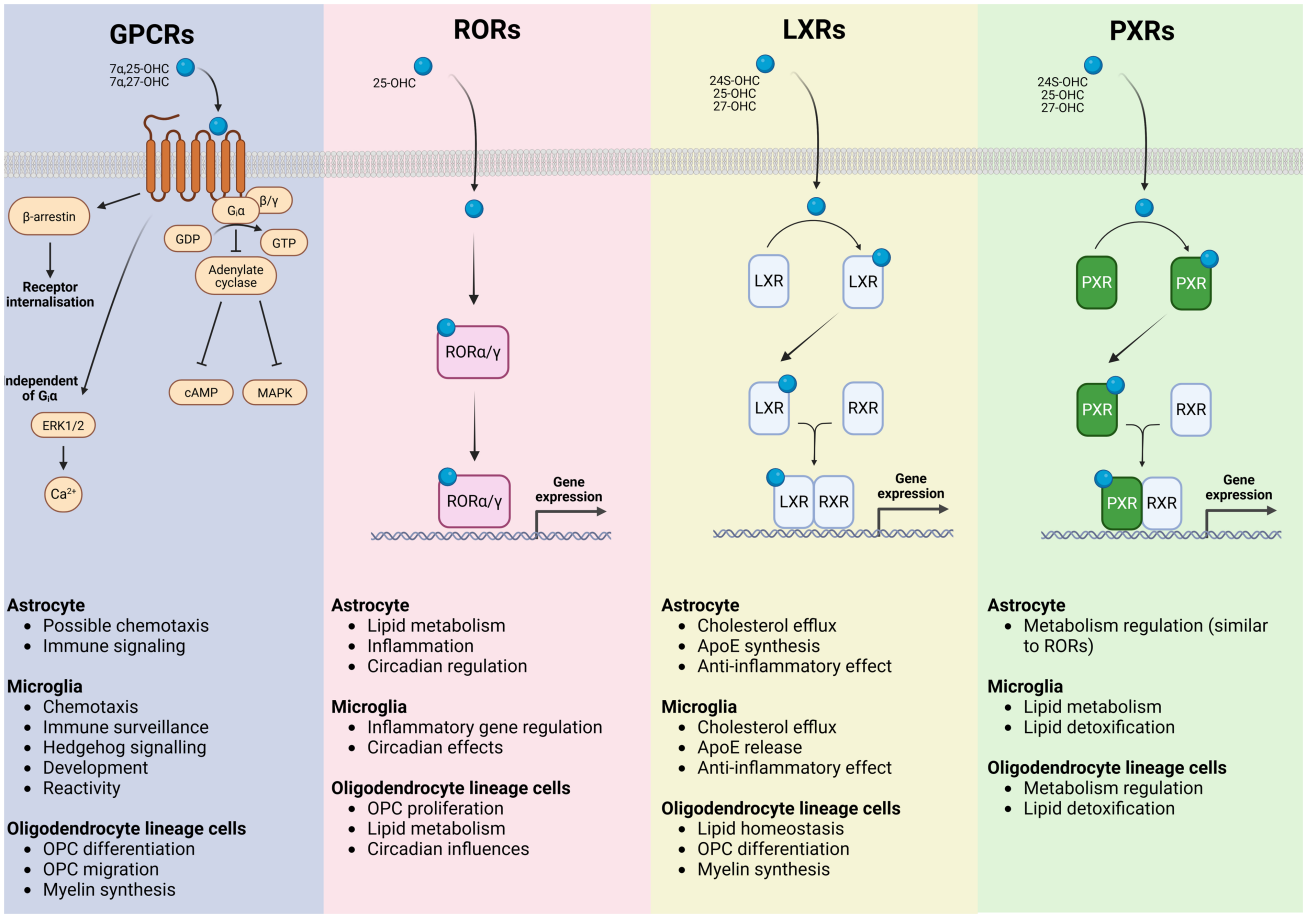
Oxysterol signaling receptors in glial cells and their downstream functional programs. Schematic representation of the major classes of oxysterol-responsive receptors expressed by astrocytes, microglia, and oligodendrocyte lineage cells, illustrating receptor-specific signaling mechanisms and the principal cellular processes they regulate. G protein–coupled receptors (GPCRs) respond to oxysterols including 7α,25-OHC and 7α,27-OHC, activating heterotrimeric G proteins and β-arrestin–dependent pathways that engage adenylate cyclase–cAMP, MAPK, ERK1/2, and Ca^2+^ signaling cascades, as well as receptor internalization. In glial cells, GPCR signaling contributes to astrocytic immune signaling and chemotactic responses, microglial chemotaxis, immune surveillance, developmental and hedgehog-related signaling, and regulation of microglial reactivity, while influencing OPC migration, differentiation, and myelin synthesis. Retinoic acid receptor–related orphan receptors (RORα/*γ*) are activated by oxysterols such as 25-OHC and function as ligand-dependent transcription factors that translocate to the nucleus to directly modulate gene expression programs controlling lipid metabolism, inflammatory tone and circadian regulation in astrocytes, inflammatory gene expression and circadian effects in microglia, and OPC proliferation, lipid metabolic pathways, and circadian influences within the oligodendrocyte lineage. Liver X receptors (LXRα/β) are activated by 24S-OHC, 25-OHC, and 27-OHC, heterodimerize with retinoid X receptors (RXRs), and bind to LXR response elements to drive transcription of genes involved in cholesterol efflux and lipid homeostasis. In astrocytes and microglia, LXR signaling promotes cholesterol export, ApoE synthesis and release and exerts anti-inflammatory effects, whereas in oligodendrocyte lineage cells it supports lipid homeostasis, OPC differentiation, and myelin synthesis. Pregnane X receptors (PXRs), activated by several oxysterols including 24S-OHC, 25-OHC, and 27-OHC also heterodimerize with RXRs to regulate transcriptional programs associated with metabolic control and xenobiotic and lipid detoxification. PXR signaling in microglia and oligodendrocyte lineage cells is linked to lipid metabolism and detoxification pathways, with astrocytic roles overlapping those mediated by RORs. Collectively, these receptor systems integrate oxysterol cues to coordinate glial lipid metabolism, inflammatory responses, and myelination, providing a mechanistic framework linking cholesterol-derived metabolites to glial functions in health and disease.

Oxysterol receptors are also expressed by cells glial cells (Figure 2), including cells of the oligodendrocyte lineage (Marinelli et al., 2016; Montani, 2021; Miralles et al., 2023) suggesting a role for oxysterol signaling in regulating these cells. Gpr17 is closely related to the Gpr183 which is known to be activated by 7α-25-DHC (Liu et al., 2011). Recent evidence suggests that 24S-OHC may act as an agonist for Gpr17 (Harrington et al., 2023). In the mouse brain, Gpr17 was found to be expressed by a subpopulation of OPCs (Miralles et al., 2023). This subpopulation of Gpr17+ OPCs displayed a transcriptomic signature distinct from Gpr17-neg OPCs, presenting enrichment towards synapse maturation and regulation of lipid metabolism (GO-term). Gpr17+ OPCs also behave differently under physiological conditions, presenting a reduced rate of differentiation compared to the overall OPC population (Miralles et al., 2023). Gpr17 receptor activation in OPCs triggers Giα interaction blocking adenylate cyclase enzyme and cyclic AMP production (cAMP). Inhibition of cAMP signaling cascade increases the expression of proteins involved in oligodendrocyte differentiation and myelination. When Gpr17 is conditionally knocked out of OPCs in the adult mouse brain, OPC proliferation is reduced while oligodendrogenesis is increased in the motor cortex (Miralles et al., 2023). In addition, a study using siRNA to silence Gpr17 reported an increase in the expression of mature OL markers and altered expression of genes involved in glucose metabolism and lipid biosynthesis (Marangon et al., 2022). Meanwhile, after a CNS insult, Gpr17+ OPCs react with proliferation and differentiation at a higher rate, supporting an important role in repair processes (Miralles et al., 2023). In contrast, Gpr17 activation in oligodendrocyte lineage cells impairs myelination. Receptor activation using Gpr17 agonist MDL29,951 (2-carboxy-4,6-dichloro-1H-indole-3-propionic acid) results in reduced myelin basic protein (MBP) expression. Gpr17 receptor activation in oligodendrocyte lineage cells triggered the Gαi/o signaling pathway, leading to a reduction in the adenylyl cyclase (cAMP) signaling cascade. Blocking cAMP signaling cascade inhibits protein kinase A (PKA) and cAMP response element-binding protein (CREB) reducing MBP expression and oligodendrocyte differentiation. In addition, Gpr17 activation also diminishes MBP expression by lessening stimulation of the exchange protein directly activated by cAMP (EPAC). The data evidence PKA and EPAC role as key downstream effectors of Gpr17 that inhibit oligodendrocyte maturation (Simon et al., 2016).

Oxysterols also interact with the nuclear LXRs, critical for lipid homeostasis in oligodendrocytes (Shackleford et al., 2017). Activation of LXRs mediated by oxysterols like 24(S),25-epoxycholesterol (24,25-EC) and 25-OHC has been shown to regulate genes involved in lipid metabolism and myelination (Shackleford et al., 2017). Oxysterols in the cell cytoplasm bind to LXR receptor resulting in dimerization with RXR subunit. LXR-RXR dimer translocate into the cell nucleus where it acts as a transcription factor regulating genes involved in OPC differentiation and myelination. Platelet-derived growth factor receptor alpha (Pdgfrα) and chondroitin sulfate proteoglycan (NG2) are markers exclusively expressed by OPC in the brain and known to be LXR-regulated. Pdgfrα gene and its regulation by LXRs may be one mechanism through which oxysterols signaling helps to control oligodendrocyte numbers in the CNS (Xu et al., 2014; Song et al., 2022). LXRα/β and their ligands in OPCs and oligodendrocytes and have been shown to promote myelination and remyelination in the cerebellum. Using a lysolecithin-induced demyelination of organotypic cerebellar slice cultures, Meffre et al. (2015) could show LXR activation enhanced oligodendrocyte maturation and remyelination. In addition, 25-OHC or synthetic TO901317 activation of LXRs induced myelin gene expression at the promoter, mRNA, and protein levels evidencing a direct transcriptional control of myelin gene expression (Meffre et al., 2015). Altogether, the findings evidenced the transcriptional control of myelin gene expression and the role of LXRs as positive modulators in central myelination and remyelination.

Oligodendrocyte lineage cells respond to oxysterol signaling via Gpr183. In mouse models, signaling via Gpr183 (or Ebi2) improved myelination and remyelination both *in vivo* (Konieczna-Wolska et al., 2024) and *ex vivo* (Rutkowska et al., 2017). Similar to Gpr17, Gpr183 activation in OPCs can potentially trigger Giα interaction inhibiting cAMP signaling cascade and increase the expression of proteins involved in oligodendrocyte differentiation and myelination (Rutkowska et al., 2017; Konieczna-Wolska et al., 2024). However, it can also act independently of Giα activating ERK1/2 pathway resulting in increased intracellular calcium signaling enhancing OPC proliferation, differentiation and myelination. Gpr183 activation can also trigger β-arresting activation and receptor internalization, reducing the signaling via Giα pathway enhancing signaling via ERK1/2 pathway. Interestingly, ERK signaling pathway is a known regulator of cell migration, especially in OPCs. As Gpr183 mediates immune cell migration (e.g., T-cell, B-cell, macrophages, eosinophils) towards oxysterol gradients (e.g., 7α,25-OHC), this could suggest a Gpr183 role in OPC chemotactic migration toward oxysterol gradients yet to be explored. It is still unclear whether Gpr183 receptor expression is restricted to a sub-population of OPCs or any specific brain regions, similar to observations from Gpr17 receptor studies in oligodendrocyte lineage cells. Pharmacological blocking of Gpr183 in cerebellar organotypic slice culture transiently delayed MBP expression staggering myelination (Rutkowska et al., 2017). Moreover, application of Gpr183 agonist 7α,25-OHC protected organotypic cerebellar slice cultures from lysolecithin (LPC) induced demyelination and reduced the levels of pro inflammatory cytokines IL1β and IL6 (Rutkowska et al., 2017). *In vivo* activation of Gpr183 using 7α,25-OHC did not enhance remyelination beyond the levels observed in spontaneously remyelinating tissue in the cuprizone demyelination model (Konieczna-Wolska et al., 2024). As 7α,25-OHC has a very short half-life when administered to mice via oral gavage (~30 min), fluorinating the endogenous 7α,25-OHC molecule to obtain the analogue CF_3_-7α,25-OHC rendered it more stable and increased its half-life to almost 10 h, allowing longer bioavailability for mice treatment. Interestingly, application of CF_3_-7α,25-OHC in the cuprizone model showed that extending the bioavailability of 7α,25-OHC is sufficient to accelerate remyelination *in vivo*. In contrast to the endogenous ligand, the analogue upregulated brain expression of GPR183 receptor and the lipid synthesis in the mouse corpus callosum. Furthermore, the CD4 + transcripts in the cerebellum and CD4 + cell number in the corpus callosum were reduced compared to vehicle-treated mice. These findings suggest a mechanism by which GPR183 / 7α,25-OHC signaling accelerates remyelination and modulates the immune response in the *in vivo* cuprizone model (Konieczna-Wolska et al., 2024). When *Gpr183* is knocked out from the mouse brain of mice that received cuprizone treatment myelin recovery is less efficient (Luxol fast blue and MBP staining) and inflammatory signaling via Abl1 and NFkB1 is altered (Klejbor et al., 2021). Moreover, Gpr183 expression and total cholesterol levels are upregulated during recovery period after cuprizone administration, potentially enhancing remyelination. *Gpr183^−/−^* mice that received cuprizone also presented attenuated loss of callosal CC1 + oligodendrocytes, suggesting a possible protective role for Gpr183 (Klejbor et al., 2021). Although there is evidence supporting the importance of oxysterols and their receptors to oligodendrocyte lineage cell function in health and disease, numerous gaps in the knowledge are found and more studies are required to fully understand how oxysterols regulate OPC and oligodendrocyte function and potentially promote myelin repair.

#### 5.1.3 Influence on myelin structure and function

Cholesterol and its derivatives are crucial for the synthesis of membranes, including myelin. They require large amounts of cholesterol for myelin membrane synthesis and structure maintenance. Oligodendrocytes are also the main players in cholesterol production in the brain, accounting for most of its biosynthesis (Dietschy, 2009; Berghoff et al., 2022; Qian et al., 2022). While oligodendrocytes are the primary source of cholesterol biosynthesis in the brain, other cells such as astrocytes and neurons also contribute to cholesterol synthesis to a lesser extent (Zhang and Liu, 2015; Qian et al., 2022). Some oxysterols can act as precursors for cholesterol biosynthesis, while others regulate cholesterol efflux or uptake. For instance, the oxysterol 24-OHC is produced within the neuron by the enzyme CYP46A1 allowing efflux from both neurons and glial cells maintaining brain cholesterol homeostasis (Björkhem, 2006; Segatto et al., 2019). 24-OHC can act as a transporter for cholesterol across the blood brain barrier. 24-OHC activates LXRs acting as transcription factor and regulating cholesterol biosynthesis (Radhakrishnan et al., 2007). Oxysterol activation of LXRs upregulate genes necessary for cholesterol synthesis and myelin formation (Shackleford et al., 2017) regulating myelin gene expression via Wnt/β-Catenin signaling pathway (Makoukji et al., 2011). Genetic ablation of LXR in the cerebellum reduced the expression of myelin basic protein (MBP), proteolipid protein (PLP) and ATP binding cassette subfamily A member 1 (ABCA1), all crucial for myelin formation and maintenance (Meffre et al., 2015; Song et al., 2022). Moreover, LXRs activation using 25-OHC greatly increased the mRNA expression of MBP, PLP and ABCA1 in primary mixed glial cell culture highlighting oxysterol vital role for myelin synthesis and maintenance (Meffre et al., 2015).

Oxysterols influence neural circuit establishment even before myelination takes place. They act during cortical development, influencing neural stem cell/progenitor behaviors and disruption of certain oxysterol metabolic pathways can lead to neurogenic defects and neurodevelopmental disorders (Tomita et al., 2022; De La Fuente et al., 2024). In mice, prior to embryonic stage E18, when the brain is not yet myelinated, CYP46A1, the enzyme that produces 24-OHC is downregulated (Wang et al., 2021). During that period, oxysterols act in oligodendrocyte lineage cell function independent of myelination - a process yet to be understood as the first wave of OPCs arise in the brain at E12. After birth CYP46A1 enzyme expression is upregulated and 24-OHC production in the brain starts - the major oxysterol in the brain after birth - coinciding with myelin genesis. Oxysterol balance keeps maintaining myelin structure throughout adulthood, playing an important role in altering lipid rafts in oligodendrocyte membranes, impacting myelin composition, fluidity and compaction. Oxysterols alter myelin directly either by increasing its fluidity causing raft destabilization, e.g., 7β-OHC or exerting stiffening promoting activity, e.g., 25-OHC (Wnętrzak et al., 2022). Therefore, oxysterols are extremely important for the establishment and maintenance of myelin, directly affecting its function in neural circuits.

Oxysterols can directly and indirectly alter action potential generation and propagation (Bezine et al., 2018b). Ion channel modulation by oxysterols leads to changes in resting membrane potential, the depolarization threshold, rate of depolarization and action potential duration. Oxysterols can also alter action potential propagation and synchronicity via modulation of myelin (Bezine et al., 2018b). Distinct localization of sterols and oxysterols in the mouse brain revealed regional cholesterol metabolism evidencing regional differences coinciding with distinct levels of myelination and consequently distinct neural circuit function (Yutuc et al., 2020). Oxysterols potentially regulate distinct regional myelination levels via nuclear receptors such as LXR (see above), regulating cholesterol turnover influencing membrane fluidity and signaling pathways. It results in regional differences in propagation and synchronicity of action potentials ultimately helping shape neural circuit formation and function.

Oxysterols can also become cytotoxic causing oxidative stress, dysfunction and death of neurons and glial cells altering neural circuits and contributing to demyelinating disorders (Björkhem et al., 2009; Bezine et al., 2018b). For instance, in multiple sclerosis (MS) and other demyelinating disorders, oxysterols may contribute to oligodendrocyte damage by promoting apoptosis and neuroinflammation. Cytotoxic oxysterols can induce caspase-independent myelin figure formation - lipid rich multilamellar cytoplasmic structures - and caspase-dependent polar lipid accumulation (Vejux et al., 2007). Myelin figure formation in the cytoplasm is a caspase-independent event closely linked with the cytotoxicity of oxysterols associated with oligodendrocyte dysfunction and neurodegeneration in demyelinating disorders. Similarly, caspase-dependent polar lipid accumulation resulting from cytotoxic oxysterols activity leads to cellular dysfunction and neurodegeneration. Local oxysterols and cholesterol metabolism also orchestrates remyelination by mediating the activity of LXR nuclear receptors, upregulating expression of genes that promote myelin genesis (Berghoff et al., 2022). The presence of elevated levels of pro-inflammatory oxysterols in the CNS correlate with increased microglial, macrophage and T-cell activation, indirectly impairing OPC differentiation and remyelination (de Freitas et al., 2022). Therefore, oxysterol metabolism dysfunction can lead to neurodegeneration associated with demyelinating disorders, altering the function of neural circuits. Moreover, it evidences the potential of oxysterol signaling pathway pharmacological targeting to prevent demyelination and consequent neurodegeneration and/or promote myelin repair.

### 5.2 Oxysterols in neuroinflammation

#### 5.2.1 Oxysterols activate neurons and astrocytes to mediate their cytotoxic and pro-inflammatory effect

While several oxysterols have been described to activate pro-inflammatory signaling and cell death pathways in neurons and astrocytes, several studies have proposed that 27-OHC and 24S-OHC are the primary mediators of these effects. Oxysterols exhibit distinct yet overlapping pro-inflammatory and cytotoxic effects in neurons, mediated through cell-specific signaling pathways. For instance, in SH-SY5Y neurons, 27-OHC activates the TGF-β/NF-κB signaling pathway, inducing TNFa production and iNOS expression, while suppressing IL-10 production (Ma et al., 2019). Additionally, another study found that 24-OHC, 27-OHC and 7β-OHC treatment in SH-SY5Y neurons leads to TLR4 activation, which in turn activates COX-2 and mPGES-1 resulting in increased expression of IL-8, MCP-1, β1-integrin, CD46 and MMP-9 (Testa et al., 2014). Apart from triggering the pro-inflammatory signaling cascade, 24S-OHC has also been shown to promote cell death signaling pathways in neurons. In SH-SY5Y neurons, 24S-OHC activates ACAT-1 (Chesnokov et al., 2021), leading to the esterification of 24S-OHC into lipid droplets. This process triggers the downstream SH- signaling of RIPK1 and the activation of necroptosis, independent of caspase-8 activation (Yamanaka et al., 2011). Apart from SH-SY5Y neurons, 27-OHC, 7β-OHC and 24S-OHC have demonstrated to upregulate CD36 and β1-integrin expressions in SK-N-BE cells, whereas only 24S-OHC induces CD36 and β1-integrin expression in NT-2 human neurons, enhancing adhesion of neuronal cells to Aβ_1–42_. Notably, only 24S-OHC potentiates the neurogenic effects of Aβ_1–42_ by increasing ROS (Gamba et al., 2011) production. This effect of 24S-OHC augmenting the neurogenic effect of Ab_1–42_ is also observed in another differentiated human neuroblastoma cell line, MSN (Ferrera et al., 2008). In astrocytes, 27-OHC triggers the TGF-β/TLR4 signaling pathway in C6 astrocytes, inducing iNOS production while suppressing TNFα, IL-1b and IL-10 production (Pfrieger and Ungerer, 2011; Ma et al., 2019). In addition, 27-OHC have also been demonstrated to induce cellular cytotoxicity by increasing ROS production and downregulating antioxidant enzymes (rGSH, GSH-Px and tSOD). This oxidative stress response is exacerbated by the suppression of Nrf2/HO-1 signaling pathway, which is required for the antioxidant response (Ma et al., 2015). These findings highlight the context-dependent effect of 27-OHC in astrocytes, where it may dampen specific pro-inflammatory cytokines while exacerbating oxidative stress response to cause cellular cytotoxicity. Beyond its role in mediating astrocyte migration, the Gpr183/oxysterol has also been implicated in astrocyte migration. In primary mouse astrocyte cultures, antagonism of Gpr183 and CYP7B1 activity using NIBR189 and clotrimazole respectively, reduced LPS-mediated migration. Although 7α,25-OHC was not directly evaluated in the study, these findings further provide evidence supporting the involvement of the Gpr183/oxysterol axis in inflammation-driven astrocyte migration (Caratis et al., 2025a). The pro-inflammatory effects of oxysterols on neurons have also been investigated *in vivo*. In C57BL/6 J mice, subcutaneous injection of 27-OHC led to increased Aβ_1-40_ and Aβ_1-42_ accumulation in the plasma and brain, accompanied by an increased TNFα, IL-6, IL-1b, and IL-17 concentrations in the brain, contributing to cognitive defects (Wang et al., 2020; Jing et al., 2025). In CYP27A1-overexpressing mice, 27-OHC was found to upregulate the expression of S100A8 and RAGE, both associated with pro-inflammatory signaling (Loera-Valencia et al., 2021). These findings were further validated e*x vivo*, where stimulation of 27-OHC in primary rat astrocytic cultures and primary cortico-hippocampal neurons increased S100A8 and RAGE expression. Mechanistically, the authors demonstrated that 27-OHC activates the RXRγ receptor to increase the accumulation of S100A8 and the subsequent expression of RAGE, contributing to the pro-inflammatory response. Collectively, these studies demonstrated that oxysterols, in particular 24S-OHC and 27-OHC contribute to neuroinflammation by promoting oxidative stress, pro-inflammatory cytokine production and cell adhesion responses.

#### 5.2.2 Oxysterols in microglia activation and migration

Oxysterols can regulate microglial activation. A board-based oxysterol screen in response to neuroinflammation revealed distinct patterns oxysterol production depending on the polarization of microglia (Mutemberezi et al., 2018). In LPS induced, M1-like BV2 microglia cells, sterol-ring oxidized oxysterols such as 4β-OHC, 5α,6β-di-OHC, 7-OHC, 7-KC were increased while side-chain oxysterols such as 25-OHC, 7α,25-OHC, 27-OHC, and 7α,27-OHC were decreased. In contrast, IL-4 induced, M2-polarized BV2 microglia cells showed minimal changes in most oxysterols, with increases observed in 7-OHCone, 7-KC, 7α,25-OHC, and 7α,27-OHC. Further supporting the role of oxysterols in microglial activation, 7-KC has been shown to activate resting BV-2 microglial cells by entering the nucleus and inducing NF-kB and Poly(ADP-ribose)-polymerase-1 (PARP-1) activation (Diestel et al., 2003). This subsequently triggers the downstream activation of iNOS, CD11a and ICAM-1. The 7-KC mediated upregulation of inflammatory and chemotactic phenotypes appears to be cell-state dependent, as 7-KC had a modest effect in amplifying the inflammatory and chemotactic phenotypes in already activated microglia cells (Diestel et al., 2003). In HMC3 microglia cells, both 25-OHC and 27-OHC have shown to activate the PI3K, ERK, and Src Signaling cascades, leading to increased IL-1β production and MHC-II activation (Son et al., 2023). These oxysterols have also shown to induce HSP60 (Kim et al., 2024), which could further contribute to IL-1β production through the activation of the TLR4/p38 MAPK signaling axis (Swaroop et al., 2016). While these studies have provided insights into oxysterol-mediated regulation of microglial activation and polarization, it does not reflect the interplay between the different cell types found within the brain microenvironment. To better reflect the cellular complexity that exists in the brain microenvironment, a study explored the pattern of oxysterol regulation in an LPS-stimulated, primary microglia-astrocytes co-culture system (Mutemberezi et al., 2018). They found that sterol-ring oxysterols like 5α,6β-di-OHC, 7-OHC, 7-OHCone were reduced while side-chain oxysterols such as 25-OHC, 24S-OHC, 7α,25-OHC, and 7α,27-OHC were increased, with the exception of 27-OHC which was found to be reduced. The authors further investigated whether addition of oxysterols to the microglial-astrocyte co-culture would regulate the pro-inflammatory response. Pre-treatment of 4β-OHC, 24(*S*),25-epoxychol, 25-OHC, and 27-OHC prior to LPS stimulation led to reduced mRNA expression of *Il6*, *Il1b*, *Mip1a*, and *Tnf* while pre-treatment of 22(R)-OHC and 24S-OHC selectively reduced *Il1b* and *Mip1a* expression and 7-OHCone reduced *Il1b* expression. The co-culture findings contrasted findings from monoculture microglial system where an opposite pattern of oxysterol production was observed highlighting the importance of cellular interactions within the brain microenvironment in regulating oxysterol metabolism. In mouse models, several studies have investigated the Gpr183/oxysterol axis in microglial activation (Romero et al., 2024; Toral-Rios et al., 2024). One study found that *Ch25h*^−/−^ mice had reduced CD68 expression in the hippocampus in response to peripheral LPS stimulation. This was further accompanied by a reduced expression of gene associated with neuroinflammation (*Trem2*, *Clec7a*, *Axl*). RNA-seq analysis of the hippocampus revealed a sex-specific difference in inflammatory regulation, where female *Ch25h*^−/−^ mice had reduced expression of genes involved in pro-inflammatory signaling and leukocyte migration. Consistently, *Ch25h*^−/−^ mice had reduced TNF and IL-6 production, accompanied by impaired NK cell and neutrophil infiltration into the hippocampus. The role of CH25H/25-OHC axis in microglial activation was similarly observed in a PS19 model of tauopathy (Toral-Rios et al., 2024) where CH25H confers protection against tau-mediated neurodegeneration. The authors found that *Ch25h*^−/−^ mice had reduced microglial activation markers in the hippocampus and entorhinal/piriform cortex, characterized by the reduced IBA1, CD68 and Clec7a expression. Bulk RNA-seq analyses and single nuclei RNA-seq of hippocampal tissue further revealed a downregulation of the pro-inflammatory pathways in the *Ch25h*^−/−^ mice. They further identified transcription factors (Rela, Nfkb1, Trp53, Sp1, Jun, and Stat3) that were differentially expressed in the *Ch25h*^−/−^ mice. These findings were corroborated by immunofluorescence analysis, which showed that microglia from *Ch25h*^−/−^ mice had reduced activation of phosphorylated p65 NF-κB and phosphorylated STAT-3. Together, these findings reinforce the critical role of the CH25H/25-OHC axis as a positive regulator of inflammatory signaling pathways in animal models of neuroinflammation.

Further demonstrating the Gpr183/oxysterol axis in regulating microglial activation, Song et al. (2024) demonstrated that CYP7B1 is critical for microglia activation in the brain. *Cyp7b1*^−/−^ mice had reduced microglial activation in the lumbar spinal cords characterized by reduced MHC-II, IBA1, Lamp2, and CD68 expression. The protective effect of CYP7B1 deficiency was found to be dependent on the myeloid cells, as EAE progression was delayed in WT mice lacking the myeloid compartment, but not in the *Cyp7b1*^−/−^ mice. Functional assays performed on primary mouse microglia and microglial-like cells from PBMCs of patients with CYP7B1 mutation further elucidated that phagocytotic activity and pro-inflammatory activation (IL-1β, IFNγ, TNFα, IL6, GM-CSF) was dependent on CYP7B1 expression (Song et al., 2024). Apart from mediating pro-inflammatory signaling, the role of Gpr183/oxysterol axis in regulating microglial migration has been demonstrated *in vivo* (Jang et al., 2016). Stereotactic administration of 25-OHC into the corpus callosum facilitated the recruitment of IBA1^+^ microglial, which was accompanied by the activation of caspase-1 in an NLRP3-dependent manner, leading to the increased IL-1β production. Taken together, these studies have highlighted the critical role of 7-KC and the Gpr183/oxysterol axis as a critical mediator of microglial activation and migration.

#### 5.2.3 Oxysterols regulate T cell migration and adhesion

In addition to mediating microglial migration into the brain during neuroinflammation, several studies have highlighted the Gpr183/oxysterol axis in regulating encephalitogenic T cell function within the brain. One study investigated oxysterol kinetics in the brain in response to different models of neuroinflammation (Mutemberezi et al., 2018). In a single dose LPS model, a modest and transient increase in 24(S)-OHC and 27-OHC was observed in the brain, peaking at 4 h post LPS stimulation and returning to baseline by 8 h. In contrast, 5α,6α-epoxychol, 5β,6β-epoxychol, 7-OHC, and 7-KC were found to decrease at 8 h post-LPS stimulation. Interestingly, 7α,25-OHC was the only oxysterol to have sustained production over the course of inflammation. This change in oxysterol levels was found to be only localized in the brain as no difference was observed in the spinal cord. The authors further went on to characterize the oxysterol kinetics during EAE. They found that 5α,6β-di-OHC, 7-KC, and 24(*S*),25-epoxychol were reduced, whereas 7a,25-OHC was increased in the brainstem over the course of EAE. These findings highlight the dynamic and context-dependent regulation of oxysterols in response to different inflammatory stimuli. Interestingly, across the LPS and EAE models (Mutemberezi et al., 2018), 7α,25-OHC was the only oxysterol observed to be increased in the brain in response to inflammation. Given its well-established role in mediating chemotaxis through Gpr183, sustained production of this oxysterol during inflammation contributes to the recruitment of immune cells during inflammation. Several studies have highlighted the role of the Gpr183/oxysterol axis in mediating immune cell recruitment during neuroinflammation. One study demonstrated that this axis plays a role in the pathogenesis of EAE by mediating the migration of encephalitogenic T cells into the CNS. The authors demonstrated that *Ch25h*^−/−^ mice had delayed disease onset accompanied by a milder disease severity. They further elucidated that both *Ch25h*^−/−^ and *Gpr183*^−/−^ mice had impaired infiltration of IL-17 producing CD44^+^CD4^+^ T cells (Th17 cells) into the CNS. Mechanistically, 7α,25-OHC mediates Th17 cells migration in a Gpr183-dependant manner (Chalmin et al., 2015). Another mechanism by which the proliferation of encephalitogenic T cells can be suppressed was demonstrated using an endothelial-specific *Ch25h*^−/−^ mice. The authors demonstrated that endothelial-specific *Ch25h*^−/−^ mice have reduced disease severity to EAE. They further elucidated that the loss of CH25H in primary mouse brain microvascular endothelial cells triggered a shift in lipid metabolism, favoring arachidonic acid metabolism and eicosanoid production. This altered lipid profile favors the expansion and recruitment of polymorphonuclear myeloid-derived suppressor cell into the brain, suppressing encephalitogenic T cell proliferation (Ruiz et al., 2023). Deletion of Cyp71b in mice (*Cyp7b1*^−/−^) similarly reduces disease onset in the EAE model, which is accompanied by milder disease severity and an impaired infiltration of Th1 and Th17 CD4^+^ T cells (Song et al., 2024).

The effect of the Gpr183/oxysterol axis in mediating T cell migration into the brain is not disease specific as in a PS19 tauopathy model, *Ch25h*^−/−^ mice had reduced T cell infiltration in the hippocampus (Toral-Rios et al., 2024). In addition to promoting T cell migration, the Gpr183/oxysterol axis enhances T cell adhesion to endothelial BBB (Caratis et al., 2025b). Using CSF from acute MS patients to mimic the neuroinflammatory environment, Gpr183 is upregulated in human brain vascular pericytes (HBPCs) while HSD3B7 expression is downregulated in the HBPCs and human brain astrocytes (HASTRs). Using a BBB spheroid model consisting of HBPCs, HASTRs and human brain microvascular ECs (HBMECs), they further elucidated that Gpr183 activation with 7α,25-OHC led to reduced VE-cadherin expression, a key component of endothelial junction integrity. This reduction in expression was reversed upon treatment with the Gpr183 antagonist, NIBR189. Similar effects were observed when BBB spheroids were incubated with CSF from MS patients, where VE-cadherin downregulation was reversed following NIBR189 treatment. Furthermore, Gpr183 antagonism reduced CD4^+^ T cell adhesion to BBB spheroids exposed to CSF of MS patients (Caratis et al., 2025b).

Taken together, these studies highlighted the role of oxysterols in regulating the diverse immune signaling pathways during inflammation – ranging from the induction of pro-inflammatory response and oxidative stress to the modulation of cell migration, demonstrating their potential as therapeutic targets in neuroinflammation.

## 6 Oxysterols and neurodegeneration

Oxysterols have emerged as critical molecular links between cholesterol metabolism, oxidative stress, and the progressive failure of neural circuits that characterize neurodegenerative disease. Under physiological conditions, brain-enriched oxysterols such as 24S-OHC support neuronal viability by regulating cholesterol turnover, synaptic plasticity and nuclear receptor signaling (Björkhem and Meaney, 2004). However, in aging and disease, the balance between enzymatically generated signaling oxysterols and non-enzymatic, oxidation-derived species becomes profoundly disrupted. Accumulation of cytotoxic oxysterols - including 7-KC, 7β-OHC and peripherally derived 27-OHC - amplifies oxidative stress, perturbs membrane architecture and initiates feed-forward cycles of lipid peroxidation and mitochondrial dysfunction. These processes converge on synaptic failure, impaired axonal integrity and ultimately neuronal loss, positioning oxysterol dysregulation as both a marker and a driver of neurodegeneration. At the synaptic level, oxysterols exert potent effects on neuronal excitability and excitotoxic vulnerability by modulating ionotropic receptor function and ion channel dynamics. Physiological concentrations of 24S-OHC act as positive allosteric modulators of NMDA receptors, facilitating synaptic plasticity and cognitive processes. In contrast, pathological elevations of 24S-OHC, 25-OHC, and 27-OHC sustain excessive calcium influx and destabilize excitatory-inhibitory balance, particularly when combined with oxysterol-induced weakening of GABAergic inhibition. These effects are compounded by oxysterol-driven disruption of cholesterol-rich membrane microdomains, leading to altered trafficking and clustering of glutamate and GABA receptors. Such synaptic dysregulation provides a mechanistic bridge between altered sterol metabolism and the early network hyperexcitability, synaptic loss and cognitive decline observed in AD, epilepsy-associated neurodegeneration and MS. Oxysterols also act as powerful modulators of glial biology, thereby shaping the inflammatory and metabolic landscape of the degenerating brain. In microglia and astrocytes, oxysterols such as 25-OHC and 27-OHC promote pro-inflammatory transcriptional programs through TLR4, NF-κB, and inflammasome signaling, while simultaneously suppressing antioxidant defenses (Pfrieger and Ungerer, 2011; Testa et al., 2014; Ma et al., 2019). Chronic exposure to these sterols biases glia toward neurotoxic phenotypes, sustaining cytokine production, reactive oxygen species generation and impaired metabolic support to neurons. In oligodendrocyte lineage cells, cytotoxic oxysterols directly induce mitochondrial damage, oxidative stress-induced autophagy/apoptosis and inflammasome-dependent cell death, compromising myelin integrity and axonal protection. Conversely, selective activation of oxysterol-responsive receptors such as LXRs and Gpr183 can promote oligodendrocyte differentiation and remyelination, underscoring the context-dependent duality of oxysterol signaling in neurodegeneration. Together, these findings position oxysterols as integrative regulators of neurodegeneration that operate across molecular, cellular and circuit scales. Rather than acting solely as by-products of oxidative damage, oxysterols actively shape disease trajectories by coordinating lipid metabolism, inflammatory signaling, synaptic function and myelin dynamics. This dual nature presents both challenges and opportunities. Indiscriminate suppression of oxysterol pathways may disrupt essential homeostatic functions, whereas pathway-specific modulation of oxysterol synthesis, metabolism or receptor engagement offers a promising strategy to decouple their physiological roles from their neurotoxic consequences. Targeting oxysterol signaling therefore represents a compelling therapeutic avenue to slow neurodegeneration, preserve circuit function and enhance endogenous repair mechanisms across a spectrum of neurological disorders.

## 7 Protective and therapeutic potential of oxysterols

Oxysterols play a complex role in the central nervous system, contributing to both cellular injury and neuroprotection depending on their type and context. Understanding and targeting oxysterol metabolism has therefore emerged as a promising strategy to mitigate toxicity, support glial health and enhance myelin repair in neurological and psychiatric disorders. Oxysterols metabolism can be modulated with the use of antioxidants, cholesterol-lowering drugs and neuroprotective agents.

Oxidative stress is a major mediator of oxysterol-induced cellular injury, making it a key target for neuroprotective interventions. Targeting oxidative stress with compounds like vitamin E, coenzyme Q10, or polyphenols may help mitigate oxysterol-induced damage. Oxysterol toxicity is closely tied to oxidative stress, making antioxidant strategies a promising therapeutic avenue (Poli et al., 2013). Compounds such as vitamin E, coenzyme Q10 and polyphenols can neutralize reactive oxygen species, thereby reducing oxysterol-driven activation of stress kinases and apoptosis (Niki, 2014; Gutierrez-Mariscal et al., 2020; Didier et al., 2023). By counteracting reactive oxygen species and preserving cellular redox balance, antioxidant therapies offer potential to safeguard neurons and glial cells from oxysterol toxicity and support overall brain health. Antioxidants may protect neurons and glia from oxysterol-induced injury and hold potential as adjunct therapies in neurodegenerative disease.

Modulating cholesterol metabolism has emerged as a promising strategy to control oxysterol levels and protect neural cells from toxicity (Wang et al., 2025). Statins and other cholesterol-modifying therapies may help regulate oxysterol levels. Cholesterol-lowering therapies offer another avenue to limit oxysterol toxicity by reducing their precursor availability. Statins and other cholesterol-modifying agents can decrease cholesterol turnover and dampen the excessive production of oxysterols. By stabilizing cholesterol homeostasis, these drugs may help reduce oxidative stress and apoptosis triggered by oxysterol accumulation, providing therapeutic benefit in conditions such as atherosclerosis and neurodegenerative disease. Importantly, because cholesterol is essential for oligodendrocyte differentiation and myelin synthesis, modulating cholesterol and oxysterol balance could protect glial health. By limiting oxysterol-driven toxicity while preserving adequate cholesterol supply, such interventions may support both neuronal survival and proper myelination. Importantly, cholesterol balance is critical for myelination, as oligodendrocyte differentiation and myelin synthesis require adequate cholesterol supply (Saher et al., 2005). Experimental studies have shown that while excessive statin treatment can impair oligodendrocyte maturation (Paintlia et al., 2005), modulation of cholesterol metabolism may reduce oxysterol toxicity while preserving glial function. Regulation of cholesterol and oxysterol balance such as through judicious use of statins can potentially reduce oxidative stress and apoptosis while supporting oligodendrocyte maturation, myelination and overall glial health.

Drugs targeting mitochondrial function and inflammation may also offer protective benefits. Neuroprotective agents that target mitochondrial dysfunction and inflammation represent another therapeutic strategy against oxysterol-induced damage. Agents such as mitochondrial stabilizers, anti-inflammatory drugs or compounds that enhance energy metabolism can help counteract oxysterol-driven oxidative stress and apoptotic signaling. For example, the mitochondria-targeted antioxidant Mito-Q has been shown to reduce oxidative damage and improve neuronal survival in models of neurodegeneration (Smith et al., 1999), while the anti-inflammatory agent minocycline protects neurons and glia by attenuating microglial activation and caspase-dependent cell death (Yrjänheikki et al., 1999; Zhu et al., 2002). By mitigating mitochondrial stress and dampening neuroinflammation, these approaches may reduce oxysterol toxicity and support long-term neuronal survival. 24S-OHC has also been proposed to exert neuroprotective effects. 24S-HC can activate liver X receptors (LXRs), nuclear receptors that regulate genes involved in cholesterol homeostasis, lipid metabolism and inflammation. Through LXR signaling, 24S-OHC can limit inflammatory responses in the brain and support neuronal and glial health. These findings evidence that certain oxysterols play a beneficial role in maintaining neural function, highlighting their potential as therapeutic modulators in neurodegenerative conditions.

Targeting oxysterol metabolism may offer new therapeutic avenues to support remyelination in conditions where myelin integrity is compromised, e.g., MS, AD, and PD. Modulating the production or signaling of specific oxysterols can potentially reduce oxidative stress, limit apoptosis and promote oligodendrocyte differentiation. Such interventions could benefit people with neurodegenerative or psychiatric disorders where enhancing glial health and myelin repair is critical for restoring neural circuit function.

## 8 Discussion, conclusion, and future directions

Oxysterols are central yet underappreciated regulators of CNS homeostasis, operating at the intersection of cholesterol metabolism, neural circuit function and neuroinflammation. Collectively, the evidence positions oxysterols not merely as by-products of cholesterol oxidation but as signaling molecules whose actions span neurons, oligodendrocyte lineage cells, astrocytes and microglia. Through convergent effects on ionotropic receptor function, membrane biophysics, nuclear receptor signaling and inflammatory cascades, oxysterols emerge as powerful modulators of excitability, myelination and vulnerability to neurodegeneration. A major conceptual advance is the dualistic nature of oxysterol signaling. Physiological oxysterols, particularly neuron-derived 24S-OHC, support synaptic plasticity, cholesterol homeostasis and myelin maintenance through tightly regulated engagement of NMDA receptors, LXRs and glial metabolic pathways. In contrast, pathological accumulation of oxysterols such as 7-KC, 25-OHC, and 27-OHC drives oxidative stress, mitochondrial dysfunction and chronic neuroinflammation. This balance between adaptive and maladaptive oxysterol signaling appears to be a critical determinant of neuronal and glial resilience across the lifespan. At the level of neural circuits, oxysterols exert profound effects by reshaping the excitation-inhibition balance. Positive allosteric modulation of NMDA receptors by 24S-OHC links cholesterol metabolism directly to synaptic plasticity and cognitive function, yet excessive or prolonged receptor potentiation - particularly in the context of elevated 27-OHC or 7-KC - may predispose circuits to excitotoxicity. In parallel, oxysterol-driven destabilization of lipid rafts alters AMPA, kainate and GABAA receptor trafficking, impairing both phasic and tonic inhibition. Such coordinated enhancement of glutamatergic drive coupled with weakened inhibitory control provides a unifying mechanistic framework for oxysterol contributions to epilepsy, neurodegeneration and neuropsychiatric disorders characterized by network hyperexcitability.

Oxysterols also integrate metabolic and inflammatory signaling through mitochondria- and redox-dependent pathways. Non-enzymatically generated oxysterols amplify reactive oxygen species production, activate stress kinases, and initiate lipid peroxidation, creating a feed-forward cycle that perpetuates neuronal and glial injury. Importantly, neurons are intrinsically vulnerable to these insults due to limited antioxidant capacity, while oligodendrocytes- highly dependent on lipid metabolism - are particularly sensitive to oxysterol-induced mitochondrial and peroxisomal dysfunction. These mechanisms align with clinical observations linking oxysterol signatures to biomarkers of neuroaxonal damage and disease severity in disorders such as MS and AD. Within the glial population, oxysterols emerge as potent regulators of neuroinflammation and myelin dynamics. Microglial and astrocytic responses to oxysterols promote cytokine production, ROS generation and altered migratory behavior, thereby shaping inflammatory microenvironments that influence neuronal survival and OPC differentiation. Notably, oxysterol-sensitive GPCRs such as Gpr17 and Gpr183 define functionally distinct OPC subpopulations and regulate key transitions between proliferation, migration and myelination. In parallel, LXR signaling links oxysterol availability to transcriptional programs controlling cholesterol transport, lipid biosynthesis and myelin gene expression. These findings collectively suggest that oxysterols serve as molecular cues coordinating immune activity, metabolic state and regenerative capacity within the CNS. Importantly, the context-dependent actions of oxysterols provide a plausible explanation for their seemingly paradoxical roles in disease. While excessive oxysterol accumulation exacerbates neuroinflammation and degeneration, appropriately timed and localized oxysterol signaling - particularly via LXR and Gpr183 pathways - can promote remyelination and circuit stabilization. This dichotomy underscores the need to move beyond viewing oxysterols solely as toxic metabolites and instead consider them as dynamic regulators whose effects depend on concentration, cellular source, receptor engagement and disease stage. From a translational perspective, targeting oxysterol metabolism and signaling presents both opportunities and challenges. Modulating enzymatic pathways that govern oxysterol synthesis, selectively engaging protective receptor pathways, or restoring physiological sterol balance may offer novel strategies to limit neurodegeneration while enhancing repair. However, given the breadth of oxysterol actions across cell types and circuits, therapeutic approaches will require precise spatiotemporal control to avoid disrupting essential homeostatic functions.

In conclusion, oxysterols constitute a pivotal molecular axis linking cholesterol metabolism to neural circuit function, glial biology and neurodegenerative disease. Acting as key mediators of oxidative stress, inflammation, mitochondrial dysfunction and protein aggregation, distinct oxysterol species orchestrate adaptive versus pathological responses across neurons and glia. Characterizing specific oxysterol profiles in normal and pathological myelination may reveal metabolic signatures that influence oligodendrocyte function and myelin integrity, providing potential biomarkers and therapeutic targets for demyelinating, neurodegenerative and neuropsychiatric disorders. However, current limitations in oxysterol quantification, including low abundance, structural diversity, and analytical sensitivity, pose challenges to accurately map their spatiotemporal dynamics in the CNS. Advances in high-resolution, cell-type–specific and regionally targeted lipidomic approaches will be critical to overcome these hurdles and fully elucidate oxysterol function. Pharmacologically modulating oxysterol signaling represents a promising strategy to enhance remyelination, support oligodendrocyte differentiation and promote myelin repair, while understanding how oxysterols intersect with other lipid signaling pathways - including sphingolipids and endocannabinoids - could further illuminate mechanisms regulating glial biology. Future studies integrating cell-type specific, regional and temporal analyses of oxysterol signaling will be essential to harness this pathway for therapeutic benefit and to advance our understanding of CNS vulnerability and resilience.
